# Comparative effects of semaglutide tirzepatide and retatrutide on renal fibrosis in UUO and aged mice

**DOI:** 10.1016/j.isci.2026.117174

**Published:** 2026-08-13

**Authors:** Mingling Ding, Xiaoxiao Li, Yuchao Wei, Jitong Wang, Bohan Jiao, Caiyue Li, Wenbo Ma, Yongbo Peng, Jinhua Shen, Guanghui Yu, Chao Gao

**Affiliations:** 1 College of Life Sciences, South-Central Minzu University, Wuhan, Hubei 430074, China; 2 The Eighth Affiliated Hospital of Southern Medical University, Foshan, Guangdong 528300, China; 3 Hubei Provincial Key Laboratory for Protection and Application of Special Plants in Wuling Area of China, College of Life Sciences, South-Central Minzu University, Wuhan, Hubei 430074, China

**Keywords:** semaglutide, tirzepatide, retatrutide, chronic kidney disease, fibrosis, inflammation

## Abstract

Chronic kidney disease is a major health burden. Given the therapeutic potential of GLP-1 receptor agonists (semaglutide, tirzepatide, and retatrutide) in diabetic nephropathy, we evaluated their anti-fibrotic and anti-inflammatory effects in non-diabetic renal fibrosis using HK-2 cells and murine UUO and aging models. Integrating *in vivo* and *in vitro* data showed model- and drug-specific patterns of action among the three agents. In the UUO model, semaglutide effects were associated with PI3K-AKT inhibition, tirzepatide effects with PI3K-AKT inhibition and PPAR pathway activation, and retatrutide effects with concurrent inhibition of both PI3K-AKT and NF-κB pathways. In the aging model, all three drugs were linked to G_2_/M arrest mitigation. Retatrutide showed the greatest efficacy at the doses tested in the models studied. This comparative study offers a framework for understanding the renoprotective actions of these drugs and generates hypotheses for future investigation.

## Highlights

• Semaglutide, tirzepatide, and retatrutide reduce renal fibrosis in UUO and aged mice
• Drug effects are associated with distinct pathways in UUO vs. aging models
• Retatrutide shows the most pronounced effects in the models tested
• G_2_/M phase arrest is a key target for anti-fibrotic action in aged kidneys

## Graphical abstract

**Figure:**
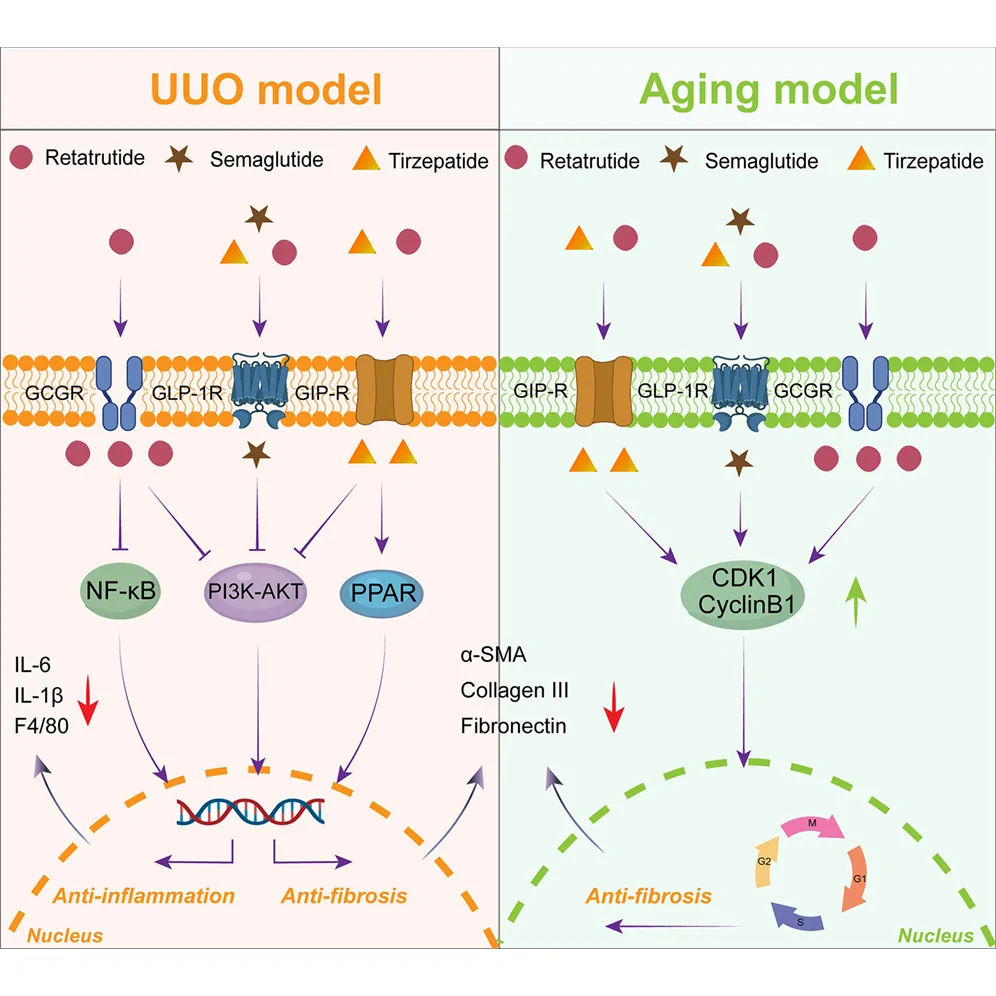

## Teaser

Fibrosis; Pharmacology

## Introduction

Chronic kidney disease (CKD) is one of the major complications of diabetes mellitus (DM), affecting approximately 40% of individuals with diabetes.^1^ CKD leads to severe renal structural and functional damage, with fibrosis and inflammation representing the primary pathological features driving disease progression and accelerating the transition to end-stage renal disease (ESRD).^2^ The pathogenic mechanisms underlying CKD are complex and heterogeneous, making therapeutic strategies that target a single pathway insufficient to effectively delay disease progression. Consequently, the development of multi-target therapeutic agents has become a critical unmet need. Glucagon-like peptide-1 (GLP-1) is a 30-amino acid peptide secreted by L-type enteroendocrine cells in the ileum and colon. GLP-1 exerts its biological effects by binding to the glucagon-like peptide-1 receptor (GLP-1R), class B G protein-coupled receptors that are widely expressed across multiple tissues.^3^ In addition to its established role in the treatment of type 2 diabetes mellitus (T2DM), GLP-1 has been demonstrated to confer protective effects in multiple organs, including the liver, heart, and kidneys.^4^^,^^5^^,^^6^^,^^7^ Studies have shown that glucagon-like peptide-1 receptor agonists (GLP-1RAs) are expressed in both the renal cortex and tubules.^8^ Accumulating evidence indicates that GLP-1RAs attenuate renal fibrosis by suppressing oxidative stress and inflammatory responses through activation of protein kinase A (PKA) or downregulation of nuclear factor-κB (NF-κB) signaling pathway,^9^^,^^10^ independently of their glycemic control effect.

Multi-target therapeutic strategies have shown enhanced efficacy in alleviating kidney disease. Combination therapy with GLP-1RAs and antihypertensive drugs, which simultaneously target the GLP-1R signaling and the renin-angiotensin system (RAS), has been shown to further ameliorate renal injury.^11^ In addition, a comprehensive evaluation of the effects of GLP-1RAs in combination with sodium-glucose cotransporter-2 inhibitors (SGLT2is) indicates that their complementary mechanisms synergistically enhance protection against both renal and cardiovascular complications in CKD.^12^ However, the concomitant use of multiple drugs inevitably increases both the economic burden and the risk of metabolic adverse effects for patients.

Against this backdrop, next-generation multi-agonist incretin-based therapies, including tirzepatide and retatrutide, have attracted considerable attention. Tirzepatide functions as a dual agonist of the GLP-1 and glucose-dependent insulinotropic polypeptide (GIP) receptors, whereas retatrutide functions as a triple agonist targeting GLP-1, GIP, and glucagon (GCG) receptors. Notably, both tirzepatide and retatrutide have shown enhanced therapeutic potential in attenuating renal injury compared with selective GLP-1RAs.^13^

Currently, research on GLP-1-based multi-target therapeutics has primarily focused on diabetic nephropathy (DN). Given that non-diabetic causes and natural aging also contribute to chronic kidney injury, we compared the renoprotective effects of a single-target agonist (semaglutide) and two multi-target agonists (tirzepatide and retatrutide) in two murine CKD models-unilateral ureteral obstruction (UUO) and aging. We evaluated their efficacy against renal fibrosis and inflammation, profiled their associated pathway signatures, and validated key observations using pathway-specific modulators. Our findings provide a reference for comparing the effects of semaglutide, tirzepatide, and retatrutide in attenuating non-diabetic renal fibrosis and inform future pathway-oriented studies.

## Results

### Semaglutide, tirzepatide, and retatrutide effectively alleviate renal function impairment in UUO mice

**Figure 1:**
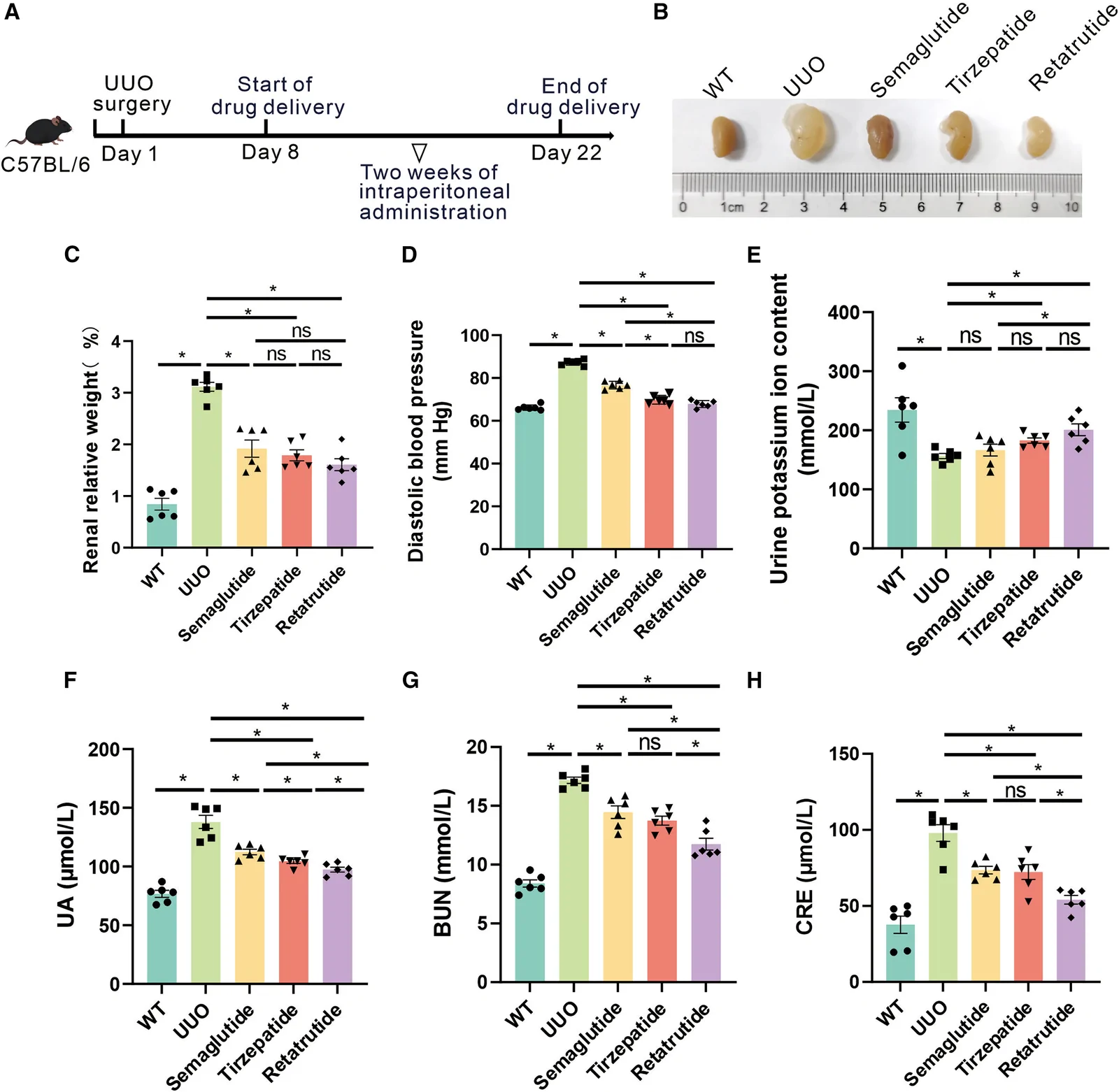
Semaglutide, tirzepatide, and retatrutide effectively alleviate renal function impairment in UUO mice (A) Schematic diagram of the UUO model experiment. (B) Comparison of kidney size and morphology among the five groups under natural light. (C) Kidney weight-to-body weight ratio. (D) Diastolic blood pressure (DBP). (E) Urinary potassium (K+) concentration. (F) Serum uric acid (UA) level. (G) Blood urea nitrogen (BUN) level. (H) Serum creatinine (CRE) level. Data are represented as mean ± SEM, significant differences were determined by one way ANOVA followed by Tukey’s post hoc test; *n* = 6, *n*, the number of independent biological replicates; ∗*p* < 0.05.

First, we examined the expression of GLP-1R, GIPR, and GCGR in the kidney to determine the direct targeting effects of the drugs on this organ (Figures S1A–S1F). Subsequently, we established a standardized drug administration regimen for the UUO model (Figure 1A). After completion of drug administration, kidney tissues were harvested for further analysis. Compared with the wild-type (WT) group, UUO mice exhibited marked renal swelling and pathological alterations, indicating successful establishment of the UUO model. In contrast, UUO mice treated with semaglutide, tirzepatide, or retatrutide showed significant attenuation of these pathological changes, indicating a protective effect of all three drugs (Figure 1B). During the treatment period, food and water intake were continuously monitored across all groups. The results showed that drug-treated UUO mice displayed a significant suppression of appetite, accompanied by a reduction in body weight. Semaglutide, tirzepatide, and retatrutide exhibited progressively enhanced weight-loss effects (Figures S1G–S1I). Structural renal alterations combined with body weight loss resulted in a significantly elevated kidney-to-body weight ratio in UUO mice compared with the WT group. Drug treatment significantly reduced this ratio; however, no statistically significant differences in restorative efficacy were observed among the three drug treatment groups (Figure 1C). Both diastolic blood pressure (DBP) and systolic blood pressure (SBP) were significantly elevated in UUO mice compared with WT controls and were markedly reduced following drug treatment (Figure 1D; Figure S1J). Notably, tirzepatide and retatrutide showed similar therapeutic effects in UUO mice, both of which were more pronounced than those observed with semaglutide. In addition, urinary potassium (K^+^) and sodium (Na^+^) levels were significantly decreased in the UUO mice. Semaglutide did not affect urinary K^+^ levels but significantly increased urinary Na^+^ levels. In contrast, both retatrutide and tirzepatide significantly elevated urinary K^+^ and Na^+^ levels; retatrutide showed superior efficacy in restoring urinary K^+^ levels compared with semaglutide, whereas no significant differences were observed among the three drugs with respect to urinary Na^+^ excretion (Figure 1E; Figure S1K). Furthermore, serum levels of uric acid (UA), blood urea nitrogen (BUN), and serum creatinine (CRE) were significantly elevated in UUO mice compared with the WT group, and all three drug treatment groups markedly reduced these parameters. Semaglutide, tirzepatide, and retatrutide progressively alleviated the increased UA, BUN, and CRE levels in UUO mice. Among the three drugs, retatrutide showed better efficacy in reducing UA, BUN, and CRE levels compared with semaglutide and tirzepatide (Figures 1F–1H). Altogether, these findings indicate that semaglutide, tirzepatide, and retatrutide significantly restore UUO-induced renal structural and functional impairment, with retatrutide exhibiting the most pronounced therapeutic effect.

### Semaglutide, tirzepatide, and retatrutide alleviate renal fibrosis and inflammation in UUO mice

**Figure 2:**
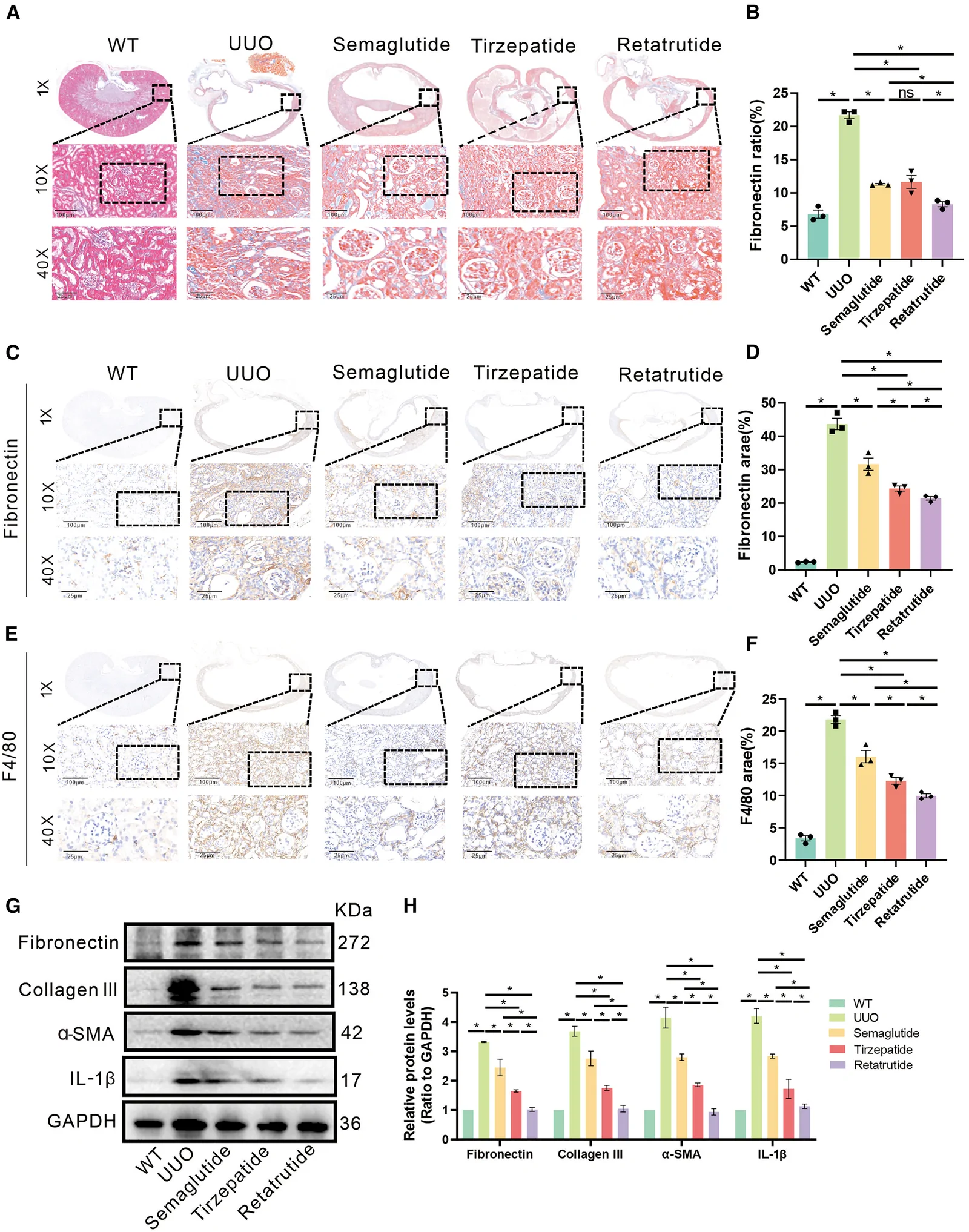
Semaglutide, tirzepatide, and retatrutide alleviate renal fibrosis and inflammation in UUO mice (A) Masson’s trichrome staining of kidney tissue. Scale bars, 100 μm (upper) and 25 μm (lower). (B) Quantitative analysis of Masson’s trichrome staining results. (C) IHC staining for fibronectin expression. Scale bars, 100 μm (upper) and 25 μm (lower). (D) Statistical analysis of fibronectin-positive area percentage. (E) IHC staining for F4/80 expression. Scale bars, 100 μm (upper) and 25 μm (lower). (F) Statistical analysis of F4/80-positive area percentage. (G) WB analysis of protein expression levels of fibronectin, Collagen III, α-SMA, and IL-1β in renal tissues. (H) Quantitative analysis of WB results. Data are represented as mean ± SEM, significant differences were determined by one way ANOVA followed by Tukey’s post hoc test; *n* = 3, *n*, the number of independent biological replicates; ∗*p* < 0.05.

Renal fibrosis, glomerular atrophy, and inflammatory responses are key features of UUO-induced kidney injury. Masson’s trichrome and hematoxylin and eosin (H&E) staining revealed a marked increase in fibrotic area and pronounced glomerular atrophy in UUO compared with controls. In contrast, treatment with semaglutide, tirzepatide, or retatrutide significantly ameliorated renal fibrosis and glomerular injury (Figure 2A; Figure S2A). Quantitative analyses further demonstrated that tirzepatide exhibited effects comparable to those of semaglutide, whereas retatrutide showed significantly greater efficacy in reducing renal fibrosis and glomerular damage than both semaglutide and tirzepatide (Figure 2B; Figure S2B). Immunohistochemistry (IHC) analysis showed significantly increased expression of Fibronectin, F4/80 and α-smooth muscle actin (α-SMA) in the renal interstitial tissue of UUO mice compared with the WT group. These elevations were significantly reduced following treatment with semaglutide, tirzepatide, or retatrutide (Figures 2C–2F; Figures S2C and S2D). Furthermore, semaglutide, tirzepatide, and retatrutide exhibited progressively stronger inhibitory effects on these markers. Consistent results were obtained from immunofluorescence (IF) staining, which showed that the expression of fibronectin, Collagen III, α-SMA, and F4/80 reduced following drug treatment (Figures S2E–S2I). Western blot (WB) analysis of renal tissues further confirmed that the protein expression of fibrotic markers, including fibronectin, Collagen III, α-SMA, as well as the inflammatory cytokine interleukin-1β (IL-1β), was significantly elevated in UUO mice compared with WT controls. These increases were markedly attenuated following treatment with semaglutide, tirzepatide, or retatrutide (Figures 2G and 2H). Comparative analyses revealed that tirzepatide showed greater inhibitory effects than semaglutide, whereas retatrutide consistently demonstrated the most pronounced suppression among the three drugs. Consistently, quantitative polymerase chain reaction (qPCR) and enzyme-linked immunosorbent assay (ELISA) analyses showed that semaglutide, tirzepatide, and retatrutide effectively reduced the expression of fibrosis-related and inflammatory factors in UUO mice (Figures S3A–S3I). *In vitro* experiments further confirmed that semaglutide, tirzepatide, and retatrutide had no toxic effects on human kidney 2 (HK-2) cells and could attenuate the transforming growth factor β (TGF-β)-induced upregulation of fibrotic and inflammatory factors in HK-2 cells (Figures S3J–S3M). Consistent with the *in vivo* findings, these drugs exhibited a similarly graded efficacy profile in suppressing fibrotic and inflammatory responses. Taken together, these results demonstrate that semaglutide, tirzepatide, and retatrutide effectively mitigate UUO-induced renal structural injury, fibrosis, and inflammation, with progressively enhanced therapeutic efficacy observed from semaglutide to tirzepatide, and retatrutide.

### In the UUO mouse model, semaglutide, tirzepatide, and retatrutide exert their therapeutic effects through modulation of distinct signaling pathways

**Figure 3:**
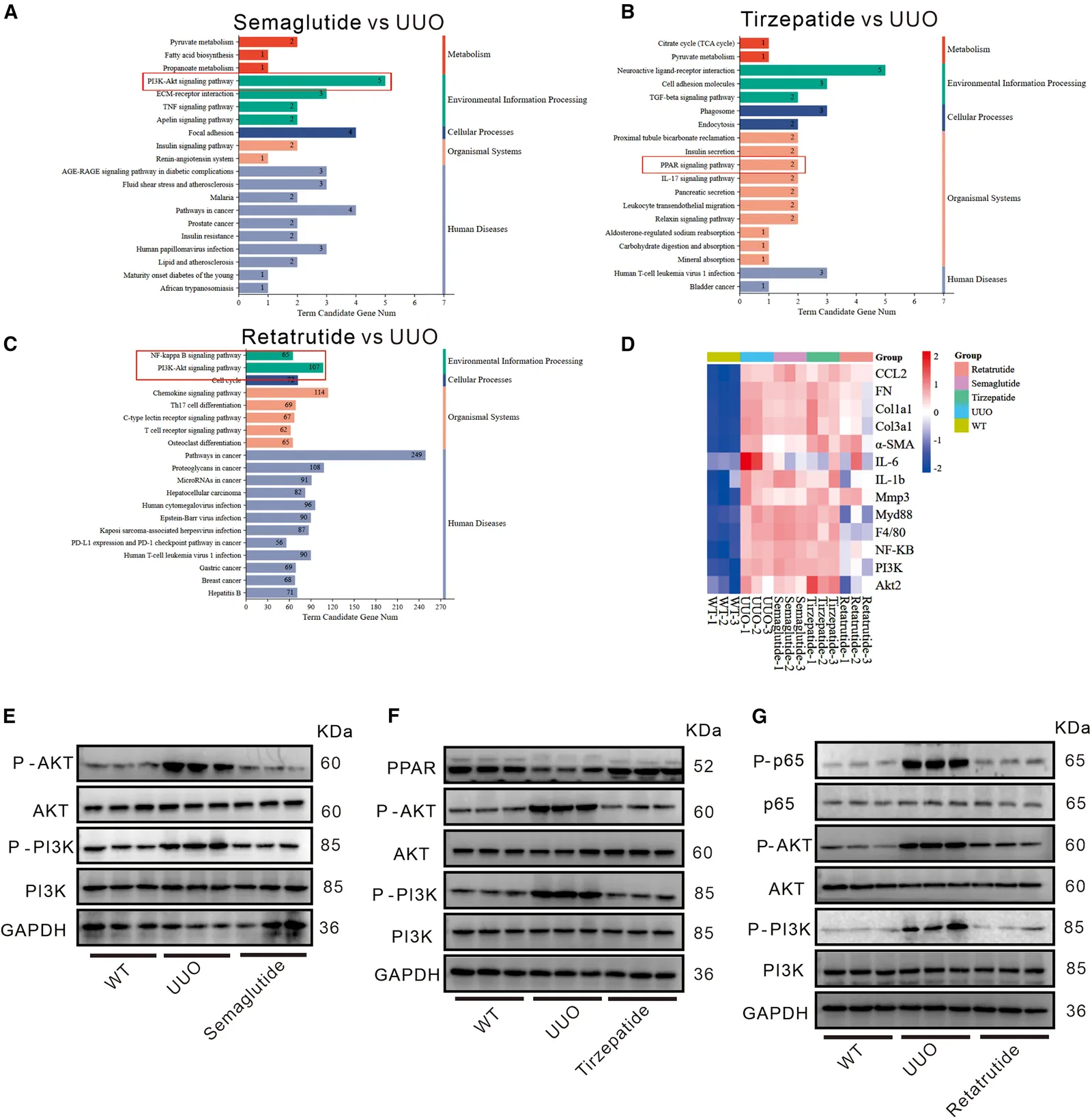
In the UUO mouse model, semaglutide, tirzepatide, and retatrutide exert their therapeutic effects by modulating different signaling pathways (A–C) KEGG pathway enrichment plots of semaglutide, tirzepatide, and retatrutide groups versus the UUO group. (D) Clustered heatmap. (E) WB analysis of PI3K-AKT pathway protein expression in renal tissues of the semaglutide group. (F) WB analysis of PI3K-AKT and PPAR pathway protein expression in renal tissues of the tirzepatide group. (G) WB analysis of PI3K-AKT and NF-κB pathway protein expression in renal tissues of the retatrutide group. Data are represented as mean ± SEM, significant differences were determined by one way ANOVA followed by Tukey’s post hoc test; *n* = 3; *n*, the number of independent biological replicates; ∗*p* < 0.05.

Based on the earlier results, we investigated whether the therapeutic effects of the drugs on renal injury in UUO mice were associated with the regulation of fibrosis- and inflammation-pathways. To further elucidate the potential mechanisms underlying drug treatment in UUO mice, we performed transcriptomic analysis of renal tissues (Figures S4A–S4F). In this analysis, the Kyoto Encyclopedia of Genes and Genomes (KEGG) pathway enrichment analysis revealed that, among the differentially expressed genes (DEGs), the phosphoinositide 3-kinase-protein kinase B (PI3K-AKT) signaling pathway was the most significantly enriched pathway in the semaglutide-treated group compared with the UUO group (Figure 3A). In the tirzepatide-treated group, KEGG enrichment analysis indicated that the peroxisome proliferator-activated receptor (PPAR) signaling pathway was predominantly associated with renal functional improvement (Figure 3B), while in the retatrutide-treated group, both NF-κB and PI3K-AKT pathways were significantly enriched and contributed to renal function restoration (Figure 3C). Clustered heatmap analysis further consistently demonstrated downregulation of fibrotic and inflammatory factors as well as related signaling pathway genes, in all the three treatment groups. Notably, the most pronounced reduction in the expression of these genes was observed in the retatrutide-treated group (Figure 3D). Phosphorylation of the PI3K-AKT signaling pathway has been reported to directly accelerate CKD progression,^14^^,^^15^ whereas PPAR signaling exerts renoprotective effects.^16^ We assessed whether UUO induction was associated with activation of the PI3K-AKT and NF-κB pathways and concomitant suppression of the PPAR pathway.

To further substantiate the pathway alterations suggested by the transcriptomic analysis, we examined the expression and activation status of key signaling pathway proteins by WB. Compared with the WT group, CKD model mice exhibited increased levels of phosphorylated PI3K (P-PI3K), phosphorylated AKT (P-AKT), and phosphorylated p65 (P-p65), accompanied by a significant reduction in PPAR expression, whereas the total protein levels of these signaling components remained comparable among groups. Relative to the UUO group, treatment with semaglutide significantly reduced the expression of P-PI3K and P-AKT (Figure 3E; Figure S5A), indicating effective suppression of PI3K-AKT pathway activation. The tirzepatide group not only downregulated P-PI3K and P-AKT but also upregulated PPAR protein expression (Figure 3F; Figure S5B). Furthermore, the results of qPCR further indicate that tirzepatide has the most significant effect in upregulating PPARβ and PPARγ (Figures S5C–S5E). In contrast, in the retatrutide group, the expression of P-PI3K, P-AKT, and P-p65 was simultaneously downregulated (Figure 3G; Figure S5F). And IF results further demonstrated that retatrutide inhibits TGF-β-induced nuclear translocation of p65 in HK2 cells (Figure S5G). Together, these findings demonstrated that the PI3K-AKT and NF-κB signaling pathways are markedly activated in the UUO model, whereas the PPAR pathway is significantly suppressed. semaglutide primarily inhibited the PI3K-AKT pathway; tirzepatide exhibited additional regulatory effects by activating the PPAR pathway, and retatrutide further suppressed the NF-κB pathway, building upon the inhibition of PI3K-AKT signaling.

### The regulatory effects of semaglutide, tirzepatide, and retatrutide on renal injury in UUO mice are closely linked to the synergistic actions of their signaling pathways

To determine whether the distinct therapeutic efficacies of semaglutide, tirzepatide, and retatrutide were mediated by their respective pathway modulation profiles, pathway-specific agonists or inhibitors were administered in combination with each drug. Specifically, the PI3K-AKT pathway agonist 740 Y-P was co-administered with semaglutide (semaglutide +740 Y-P); the PPAR pathway inhibitor GW9662 was combined with tirzepatide (tirzepatide + GW9662); and the NF-κB pathway agonist R848 was administered together with retatrutide (retatrutide + R848). Additionally, to examine potential synergistic or cooperative interactions between signaling pathways, tirzepatide-treated mice received combined administration of 740 Y-P and GW9662 (tirzepatide +740 Y-P + GW9662), whereas retatrutide-treated mice received combined administration of 740 Y-P and R848 (retatrutide +740 Y-P + R848).

**Figure 4:**
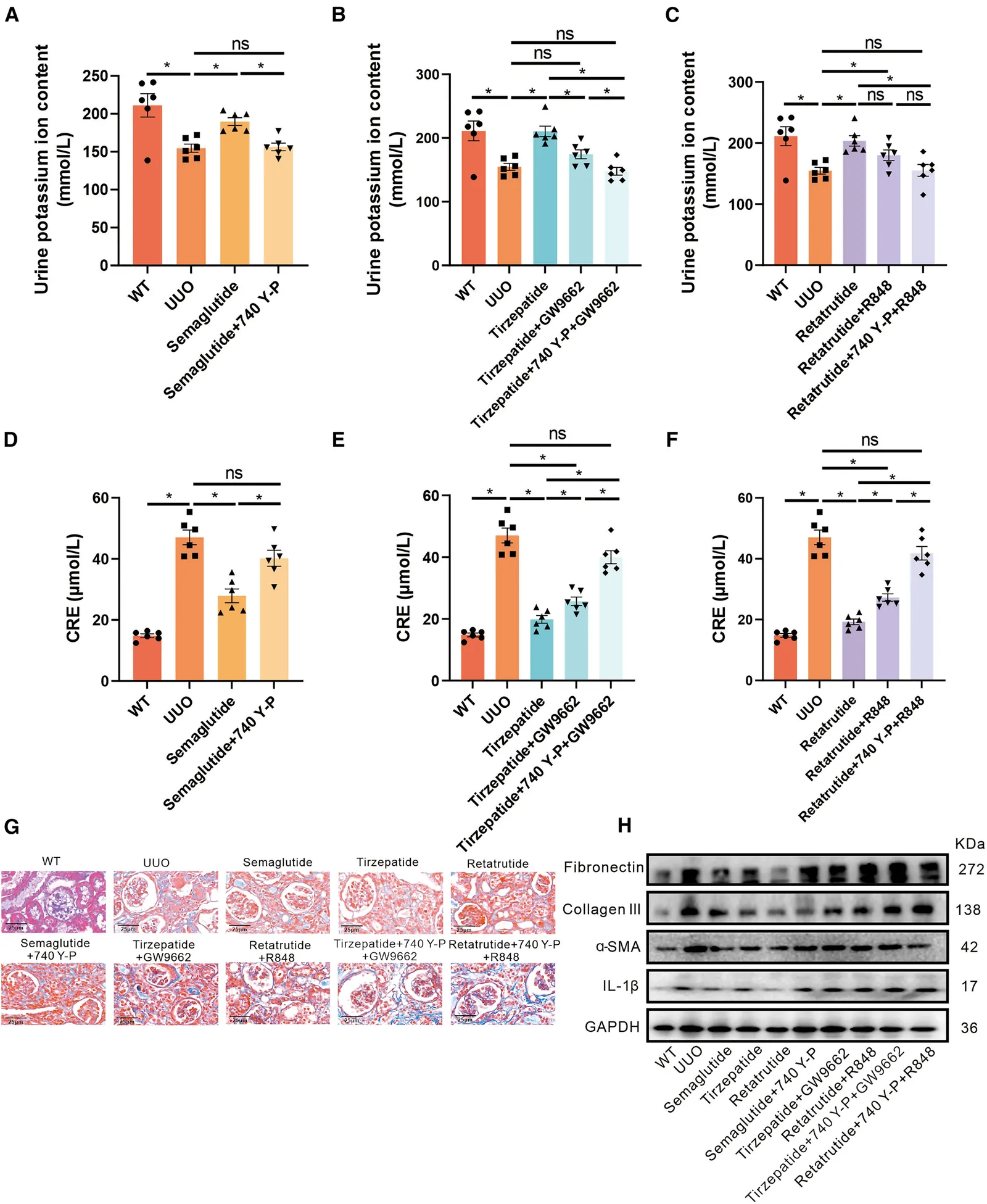
The regulatory effects of semaglutide, tirzepatide, and retatrutide on renal injury in UUO mice are closely linked to the synergistic actions of their signaling pathways (A–C) Urinary K+ levels in the semaglutide, tirzepatide, and retatrutide groups before and after administration of pathway agonists or inhibitors (*n* = 6). (D–F) Changes in CRE levels in the semaglutide, tirzepatide, and retatrutide groups before and after administration of pathway agonists or inhibitors (*n* = 6). (G) Masson’s trichrome staining of kidney sections from each group before and after administration of pathway agonists or inhibitors (*n* = 3). Scale bars, 25 μm. (H) WB analysis of fibronectin, Collagen III, α-SMA, and IL-1β protein expression levels in renal tissues of each group before and after administration of pathway agonists or inhibitors (*n* = 3). Data are represented as mean ± SEM, significant differences were determined by one way ANOVA followed by Tukey’s post hoc test; n: the number of independent biological replicates, ∗*p* < 0.05.

Urinalysis revealed that co-administration of single pathway agonists or inhibitors partially attenuated the restorative effects of semaglutide and tirzepatide on urinary K^+^ and Na^+^ levels compared with their respective monotherapy groups. In the retatrutide-treated group, although regulatory efficacy declined after R848 administration, no statistically significant difference was observed relative to the treatment group. Notably, combined administration of dual pathway agonists or inhibitors almost completely abolished the regulatory effects of tirzepatide and retatrutide on urinary K^+^ and Na^+^ levels (Figures 4A–4C; Figures S6A–6C). Consistently, assessment of CRE, UA, and BUN demonstrated that dual treatment exerted stronger inhibitory effect on the therapeutic efficacy of tirzepatide and retatrutide than treatment with a single agonist or inhibitor (Figures 4D–4F; Figures S6D–S6I). Furthermore, renal histopathological staining and WB analysis similarly indicated that dual pathway modulation markedly counteracted the renoprotective and anti-fibrotic effects of the drugs, restoring the renal fibrosis area, the protein expression levels of fibrotic markers (fibronectin, Collagen III, α-SMA), and the inflammatory cytokine IL-1β, to levels comparable to those observed in the UUO group (Figures 4G and 4H; Figures S7A–S7G, and S8A–S8C).

Collectively, these results demonstrated that, in the UUO mouse model, semaglutide, tirzepatide, and retatrutide protected renal function through modulation of distinct signaling pathways: semaglutide primarily inhibited the PI3K-AKT pathway; tirzepatide combined PI3K-AKT inhibition with activation of the PPAR pathway; and retatrutide concurrently inhibited both PI3K-AKT and NF-κB pathways. The different therapeutic efficacy among the three drugs might be attributed to synergistic modulation of multiple signaling pathways.

### Semaglutide, tirzepatide, and retatrutide alleviate renal function impairment in aged mice

**Figure 5:**
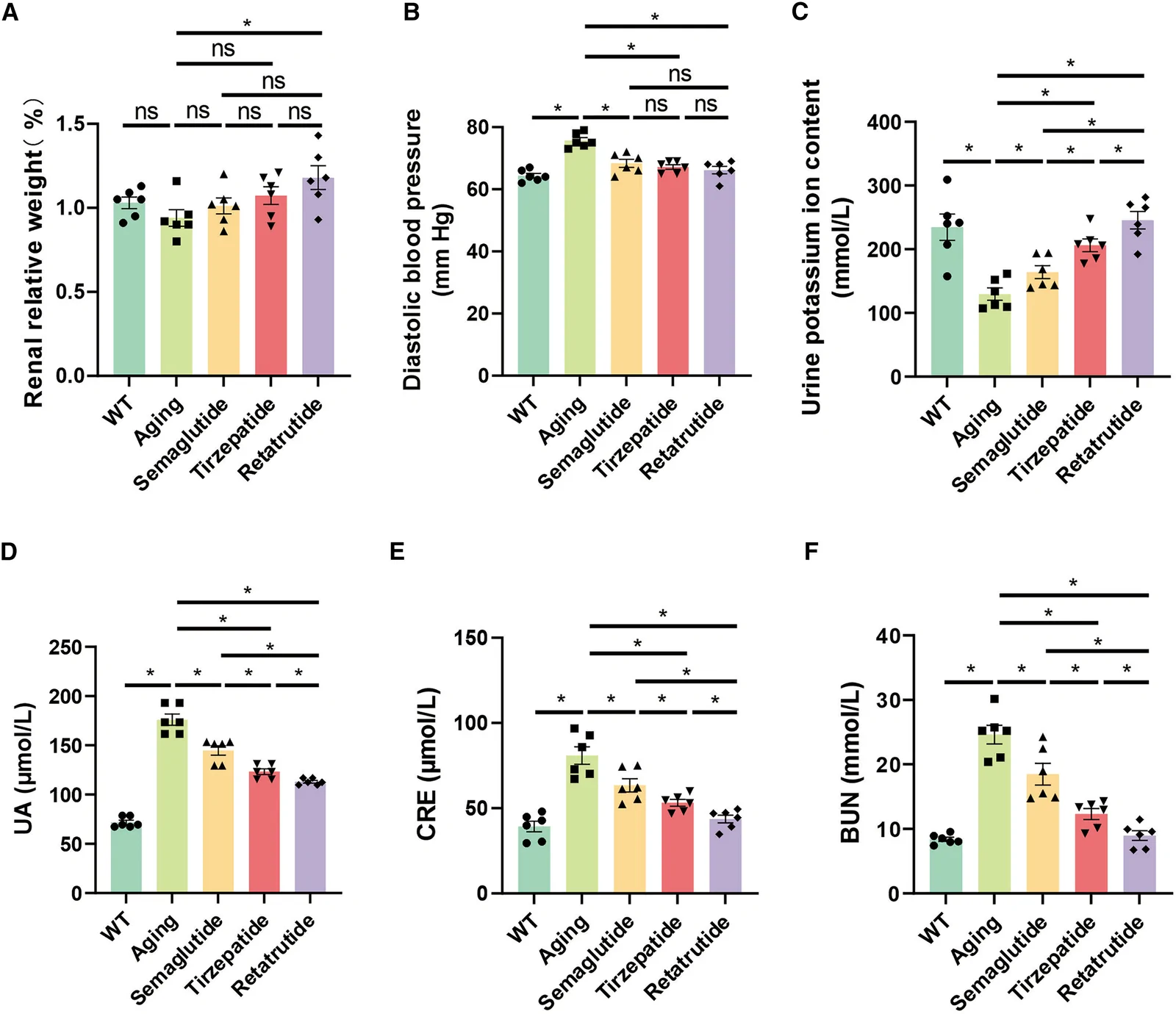
Semaglutide, tirzepatide, and retatrutide alleviate renal function impairment in aged mice (A) Kidney weight-to-body weight ratio. (B) Diastolic blood pressure (DBP). (C) Urinary K+ concentration. (D–F) Changes in serum UA, CRE, BUN levels. Data are represented as mean ± SEM, significant differences were determined by one way ANOVA followed by Tukey’s post hoc test; *n* = 6; *n*, the number of independent biological replicates; ∗*p* < 0.05.

Aging is associated with progressive degenerative changes across multiple organ systems. Recent studies have shown that GLP-1RAs can counteract systemic functional decline induced by aging^17^; however, their regulatory effects on age-associated renal impairment remain incompletely characterized. CKD is a common complication of aging, and senescent kidneys share similar pathological and clinical manifestations with CKD.^18^^,^^19^ This finding is consistent with our analysis of senescence and renal injury markers (p21, KIM-1, and IL-6), further confirming the correlation between senescence and renal injury (Figures S9A–S9C). To investigate whether semaglutide, tirzepatide, and retatrutide exert therapeutic effects on aging-induced renal injury and to compare their relative efficacy, an aging mouse model was employed. Consistent with observations in the UUO model, treatment with semaglutide, tirzepatide, or retatrutide significantly suppressed food and water intake and resulted in a reduction in body weight in aged mice (Figures S9D–S9F). Compared with the WT group, aged mice exhibited a decreased kidney-to-body weight ratio. This reduction was partially restored after drug treatment, with the retatrutide-treated group showing a statistically significant restoration (Figure 5A). In addition, both DBP and SBP were significantly elevated in aged mice relative to WT controls and were markedly reduced after drug treatment (Figures 5B; Figure S9G). With respect to SBP regulation, tirzepatide and retatrutide demonstrated superior efficacy compared with semaglutide, with similar effects between the two. However, no statistically significant differences in efficacy were observed among the three drugs in modulating DBP.

Measurements of urinary K^+^ and Na^+^ levels showed that both were significantly decreased in aged mice compared with the WT controls, whereas treatment with drugs effectively restored these ion levels (Figures 5C; Figure S9H). Tirzepatide exhibited greater efficacy than semaglutide, while retatrutide showed superior restorative effects on urinary K^+^ levels compared with tirzepatide, though no significant difference was observed between the two drugs in regulating urinary Na^+^ excretion. Consistently, assessment of renal function markers revealed that drug treatments significantly reduced UA, CRE, and BUN levels in aged mice. Notably, semaglutide, tirzepatide, and retatrutide showed a clear graded efficacy in modulating UA, CRE, and BUN, with tirzepatide demonstrating greater efficacy than semaglutide, and retatrutide showing the most pronounced effect (Figures 5D–5F). Taken together, these results indicated that semaglutide, tirzepatide, and retatrutide provide significant renal protection in aged mice, with progressively enhanced therapeutic efficacy across the three drugs.

### Semaglutide, tirzepatide, and retatrutide reduce renal fibrosis in aged mice

**Figure 6:**
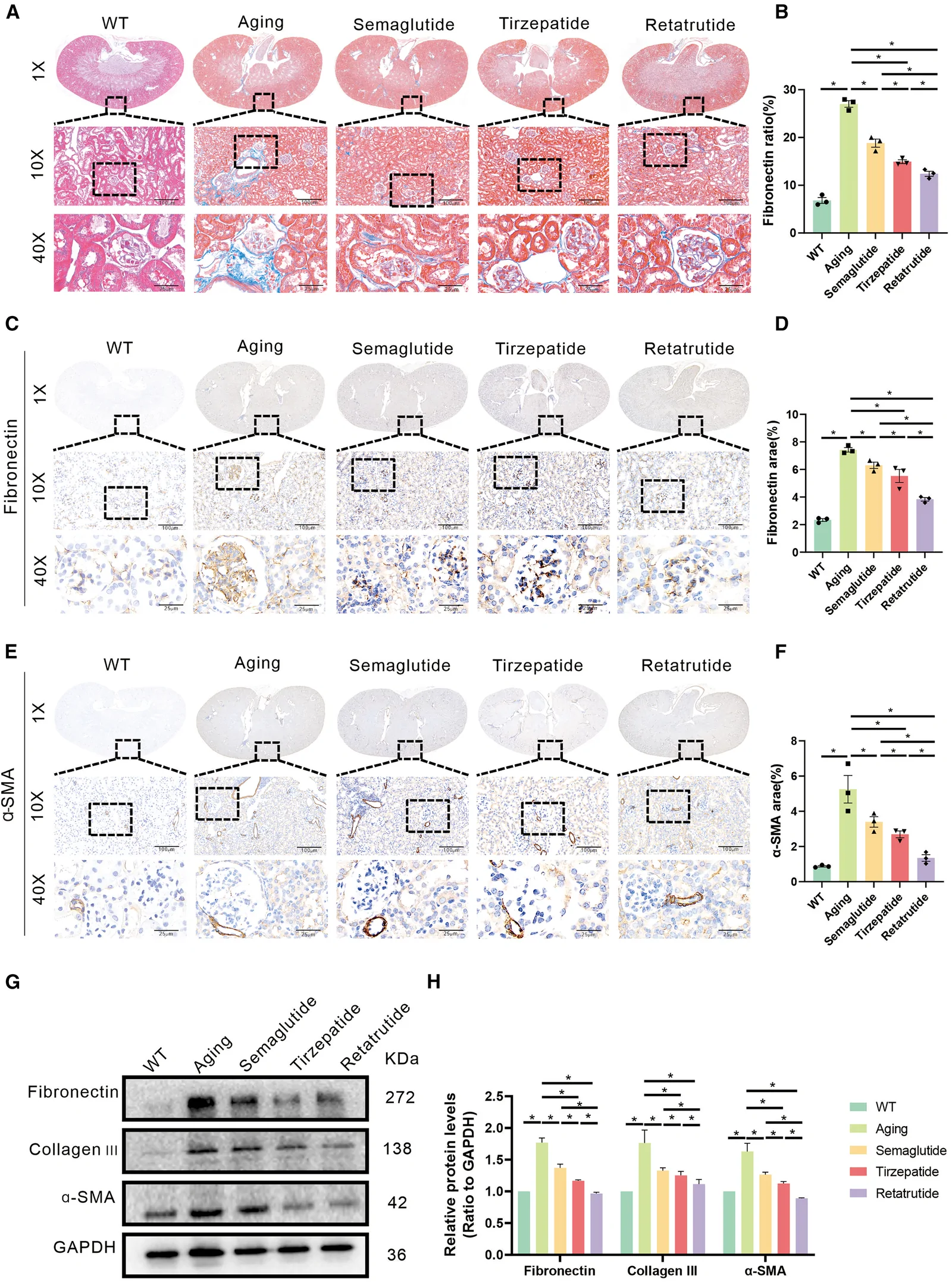
Semaglutide, tirzepatide, and retatrutide reduce renal fibrosis in aged mice (A and B) Masson’s trichrome staining of kidney tissue and quantitative analysis of positive area. Scale bars, 100 μm (upper) and 25 μm (lower). (C–F) IHC staining for fibronectin and α-SMA expression with statistical analysis of positive area percentage. Scale bars, 100 μm (upper) and 25 μm (lower). (G and H) WB analysis of fibronectin, Collagen III, and α-SMA expression with quantitative statistics. Data are represented as mean ± SEM, significant differences were determined by one way ANOVA followed by Tukey’s post hoc test; *n* = 3; *n*, the number of independent biological replicates; ∗*p* < 0.05.

Focal segmental glomerulosclerosis (FSGS), tubular atrophy, and interstitial fibrosis are major pathological features of aging kidneys, with disease severity positively correlated with advancing age.^20^^,^^21^^,^^22^ Histological staining of renal tissues revealed significantly exacerbated fibrosis and severe glomerular shrinkage in aged mice compared with the WT group. In contrast, treatment with semaglutide, tirzepatide, or retatrutide significantly reduced renal fibrosis and ameliorated glomerular lesions in the aged mice. The antifibrotic efficacy of these drugs exhibited a graded pattern, with retatrutide demonstrating the most pronounced effect, followed by tirzepatide and semaglutide (Figures 6A and 6B; Figures S10A and S10B). IHC analysis demonstrated markedly elevated expression of fibronectin and α-SMA in aged mice, which were significantly reduced after drug intervention. Quantitative analyses further revealed a graded antifibrotic efficacy among the three agents, with therapeutic effects increasing progressively from semaglutide to tirzepatide and retatrutide (Figures 6C–6F). Consistently, IF staining confirmed the downregulation of fibronectin, Collagen III, and α-SMA expression in response to all three treatments (Figures S10C–S10F). Moreover, WB and qPCR analyses further validated these findings, demonstrating significant reduction in fibrotic marker expression at both the protein and transcript levels after drug treatment, and retatrutide exhibited the strongest anti-fibrotic effect among the three drugs (Figures 6G and 6H; Figures S10G–S10I).

### Semaglutide, tirzepatide, and retatrutide alleviate renal fibrosis in aged mice by modulating the G_2_/M phase of the cell cycle

**Figure 7:**
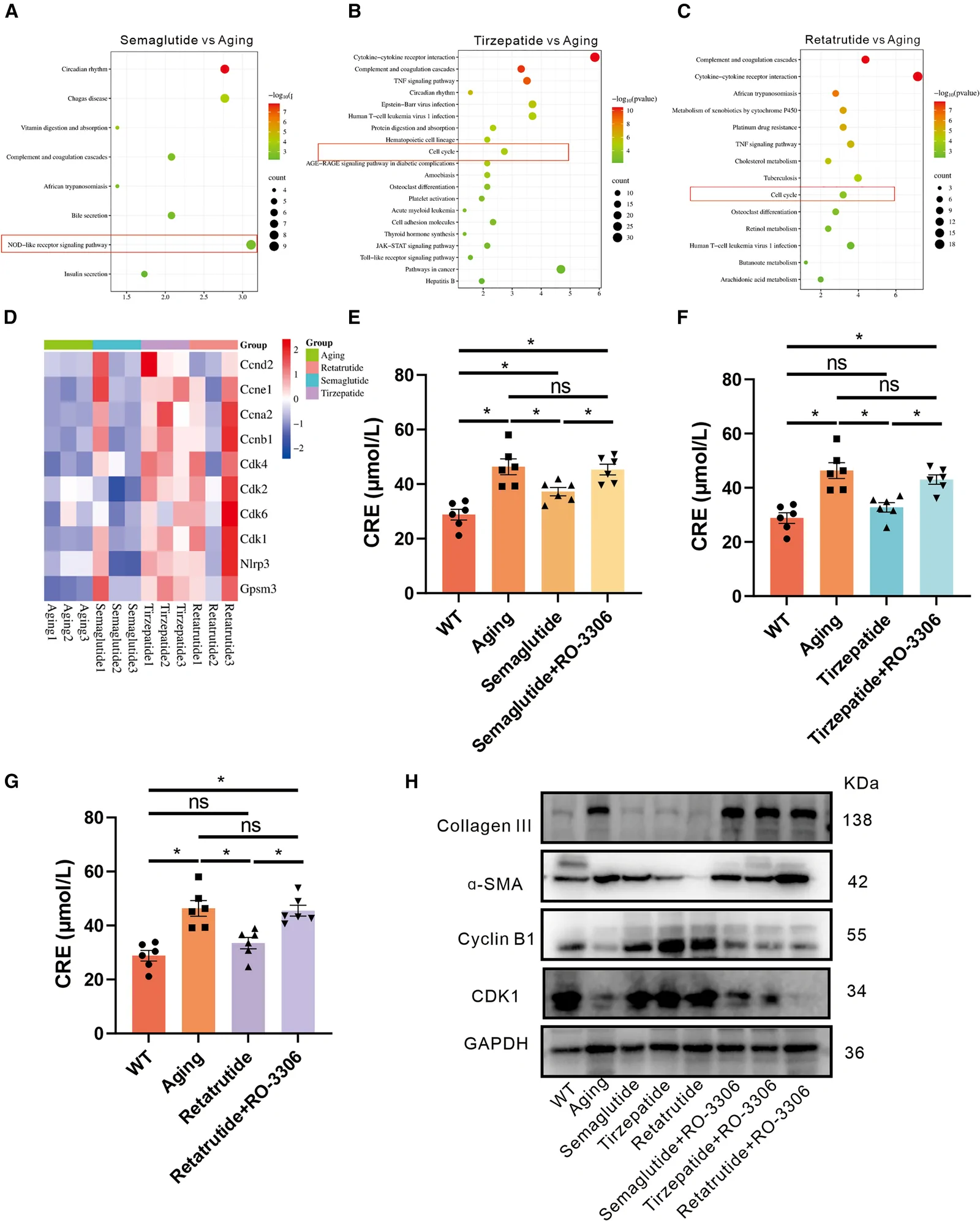
Semaglutide, tirzepatide, and retatrutide alleviate renal fibrosis in aged by modulating the G2/M phase (A–C) KEGG pathway enrichment plots of semaglutide, tirzepatide, and retatrutide groups versus the aged group (*n* = 3). (D) Clustered heatmap (*n* = 3). (E–G) CRE levels in the semaglutide, tirzepatide, and retatrutide groups before and after inhibitor treatment (*n* = 6). (H) WB analysis of Collagen III, α-SMA, Cyclin B1, and CDK1 protein expression levels in renal tissues of each group before and after inhibitor treatment (*n* = 3). Data are represented as mean ± SEM, significant differences were determined by one way ANOVA followed by Tukey’s post hoc test; *n*, the number of independent biological replicates, ∗*p* < 0.05.

To elucidate the potential mechanisms of semaglutide, tirzepatide, and retatrutide in aged mice, we performed RNA sequencing (RNA-seq) analysis of renal tissues from aged mice (Figures S11A–S11F). KEGG pathway enrichment analysis revealed that the DEGs in the semaglutide-treated group compared with the aged group were predominantly enriched in the non-obese diabetic (NOD)-like receptor signaling pathway, while DEGs identified in both the tirzepatide- and retatrutide-treated groups were significantly enriched in the cell cycle pathway (Figures 7A–7C). Notably, these pathway-targeted DEGs consistently included cyclin-dependent kinase 1 (CDK1) and Cyclin B1, both of which are critical regulators of the G_2_/M phase transition (Figures S12A–S12C). Consistent with these findings, clustered heatmap analysis demonstrated upregulation of cell cycle-associated genes in the drug-treated groups (Figure 7D). The cell cycle regulators CDK1 and Cyclin B1 act as key drivers of the G_2_/M phase transition. Given previous reports demonstrating that G_2_/M phase arrest promotes renal inflammation and fibrosis,^23^^,^^24^ we examined whether the anti-fibrotic effects of semaglutide, tirzepatide, and retatrutide in aged mice were associated with enhanced progression through the G_2_/M phase of the cell cycle.

To validate the sequencing findings, we administered the G_2_/M phase specific inhibitor RO-3306 intraperitoneally (i.p.) to aged mice in combination with each drug treatment. Assessment of renal function markers showed that RO-3306 completely abolished the therapeutic effects of all three drugs on CRE (Figures 7E–7G), BUN (Figures S13G–S13I), and UA (Figures S14A–S14C). Consistently, measurements of urinary Na^+^ and K^+^ levels revealed that RO-3306 treatment reversed the renoprotective effects of all three drugs on electrolyte homeostasis (Figures S13A–S13F). WB analysis of renal tissues revealed that drug treatment significantly reduced the expression of Collagen III and α-SMA, while concomitantly increasing the expression of CDK1 and Cyclin B1. Notably, tirzepatide and retatrutide induced more pronounced upregulation of CDK1. In contrast, administration of RO-3306 markedly elevated Collagen III and α-SMA expression and suppressed CDK1 and Cyclin B1 levels (Figure 7H; Figures S14D–S14F). Collectively, these results demonstrate a critical role for cell cycle regulation in aging-associated renal dysfunction and indicate that modulation of the G_2_/M phase contributes to the renoprotective effects of semaglutide, tirzepatide, and retatrutide in aged mice.

### Semaglutide, tirzepatide, and retatrutide suppress senescence-induced fibrotic responses in HK-2 cells

**Figure 8:**
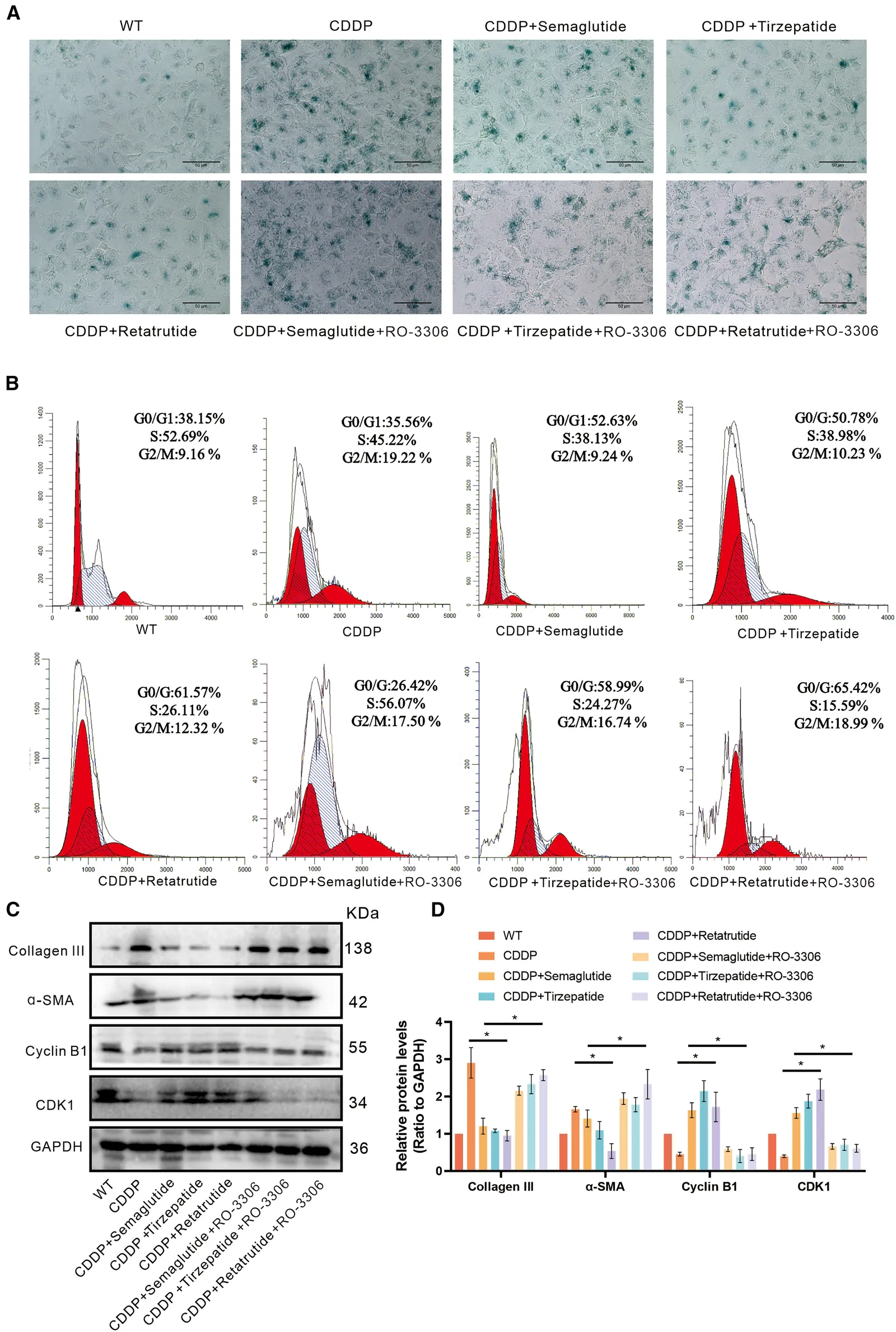
Semaglutide, tirzepatide, and retatrutide inhibit the increase in fibrotic levels induced by senescence in HK-2 cells (A) β-galactosidase staining. Scale bars, 50 μm. (B) Flow cytometric analysis of HK-2 cell cycle. (C) WB detection of the expression of Collagen III, α-SMA, Cyclin B1 and CDK1 in HK-2 cells. (D)Statistical graphs of WB analysis showing Collagen III, α-SMA, Cyclin B1, and CDK1 expression in HK-2 cells across experimental groups before and after inhibitor treatment. Data are represented as mean ± SEM, one-way ANOVA followed by Tukey’s post hoc test was used for comparisons involving five or more groups; *n* = 3; *n*, the number of independent biological replicates; ∗*p* < 0.05.

To further examine the regulatory effects of semaglutide, tirzepatide, and retatrutide on G_2_/M phase arrest in a cellular senescence model, HK-2 cells were subjected to cisplatin (CDDP)-induced senescence and treated with the three drugs, with or without co-administration of the G_2_/M phase inhibitor RO-3306. Senescence-associated β-galactosidase staining demonstrated that treatment with semaglutide, tirzepatide, or rRetatrutide significantly reduced the proportion of senescent cells, whereas the addition of RO-3306 markedly increased senescent cell prevalence (Figure 8A; Figures S15A–S15C). Flow cytometry analysis demonstrated a significantly increased proportion of cells arrested in the G_2_/M phase in the senescence model group, indicating G_2_/M phase cell-cycle arrest. Drug treatment markedly reduced the percentage of G_2_/M phase-arrested cells, consistent with promotion of G_2_/M phase progression. In contrast, co-treatment with RO-3306 abolished the cell cycle-modulating effects of semaglutide, tirzepatide, and retatrutide (Figure 8B; Figures S15D–S15F). WB analysis showed that RO-3306 suppressed the drug-induced upregulation of CDK1 and Cyclin B1 protein expression. Additionally, the three drugs significantly attenuated the expression of fibrosis-related proteins (Collagen III and α-SMA) in senescent HK-2 cells; these antifibrotic effects were effectively reversed by RO-3306 (Figures 8C and 8D). Together, these findings indicate that modulation of the G_2_/M phase progression is a key mechanism underlying the protective effects of semaglutide, tirzepatide, and retatrutide against cellular senescence-associated fibrotic responses.

## Discussion

CKD represents a substantial global health burden, affecting more than 850 million individuals worldwide.^25^^,^^26^ The progression of CKD is driven primarily by two interrelated pathological processes: inflammation and fibrosis, which together accelerate renal structural damage and functional decline.^27^^,^^28^ Emerging evidence indicates that these processes are closely associated with dysregulated cell cycle control in renal epithelial cells; aberrant cell-cycle arrest can directly lead to tissue fibrosis.^29^^,^^30^ Consequently, therapeutic strategies aimed at mitigating inflammation, fibrosis, and their upstream regulatory mechanisms are critical for preserving renal function. In this context, GLP-1RAs have attracted considerable attention due to their demonstrated capacity to alleviate renal inflammation and glomerular injury, highlighting their potential renoprotective properties.^31^ In this study, combining *in vivo*, *in vitro*, and transcriptomic analyses, we evaluated and compared the therapeutic effects of three GLP-1RA family members—semaglutide, tirzepatide, and retatrutide-in UUO and aging mouse models. Our study focused on preliminarily exploring the anti-inflammatory and anti-fibrotic mechanisms underlying the renoprotective effects of these agents. The results revealed distinct, drug-specific pathway profiles for the three agents in the UUO model: semaglutide effects were associated primarily with PI3K-AKT inhibition; tirzepatide effects were associated with PI3K-AKT inhibition together with PPAR pathway activation; and retatrutide effects were associated with concurrent inhibition of both PI3K-AKT and NF-κB pathways. In the aging model, all three drugs were linked to mitigation of G_2_/M arrest. Notably, retatrutide showed relatively more pronounced effects in mitigating renal inflammation and fibrosis in both models at the doses tested.

Semaglutide, tirzepatide, and retatrutide have been reported to delay gastric emptying, increase satiety, reduce food intake, and induce significant weight loss.^32^^,^^33^^,^^34^ Consistently, our study showed that all three drugs reduced food and water intake and promoted weight loss in both UUO and aging mouse models. In the UUO model, treatment with semaglutide, tirzepatide, or retatrutide effectively suppressed renal swelling and progressively decreased kidney weight, in parallel with overall body weight loss. As a result, no significant differences in the kidney-to-body weight ratio were observed among the treatment groups. In aged mice, drug treatment did not significantly alter absolute kidney weight but led to a reduction in body weight, thereby resulting in an upward trend in the kidney-to-body weight ratio. Admittedly, reduced food intake and body weight loss can lead to alterations in systemic metabolism and physiological function, and may even directly affect kidney function. However, the renoprotective effects of GLP-1RAs have been demonstrated to be independent of their benefits on weight loss, blood pressure reduction, or glycemic control.^35^^,^^36^ Moreover, our *in vitro* experiments confirmed that all three drugs directly suppress TGF-β-induced fibrosis and cisplatin-induced senescence-associated fibrosis. Even so, the contribution of caloric or metabolic changes to renal function cannot be completely excluded.

Hypertension is both a major driver of CKD progression and an inevitable consequence of CKD; therefore, maintaining stable blood pressure is a crucial component of CKD management.^37^ Consistent with previous reports that GLP-1RAs improve blood pressure in patients with type 2 diabetes,^38^ all three drugs significantly reduced blood pressure in both models. In the UUO mice, tirzepatide and retatrutide showed more pronounced effects than semaglutide, although no significant difference was observed between tirzepatide and retatrutide. This enhanced efficacy may be attributed to their broader pathway modulation, including activation of PPAR signaling, which has been implicated in suppression of angiotensin activity, and inhibition of NF-κB-mediated inflammatory signaling, both of which are known contributors to hypertension.^39^^,^^40^ In aged mice, the relative anti-hypertensive efficacy of the three drugs on blood pressure were less consistent. This variability likely reflects the complex pathophysiology of aging-associated hypertension, which involves not only renal dysfunction but also dysregulation of the nervous system and the renin-angiotensin-aldosterone system (RAAS).^41^

Renal tissues are essential for maintaining electrolyte and acid-base homeostasis. In patients with kidney disease, a reduced glomerular filtration rate impairs urinary excretion of K^+^ and Na^+^, thereby triggering compensatory mechanisms such as aldosterone stimulation and enhanced intestinal K^+^ excretion to preserve systemic electrolyte homeostasis.^42^ Consistent with this, urinary K^+^ and Na^+^ levels were reduced in UUO and aged mice compared with controls. This reduction may, at least in part, reflect increased water intake and altered urine volume in these models, leading to dilution of urinary electrolyte concentrations. Drug treatment partially restored urinary K^+^ and Na^+^ levels, which may reflect renal injury-associated alterations in the intestinal microenvironment that influence incretin signaling, including enhanced basal synthesis and release of GLP-1.^43^ During GLP-1 secretion, intestinal epithelial cells activate adenosine triphosphate (ATP)-sensitive potassium channels, which may contribute to systemic electrolyte regulation and potassium handling.^44^

In line with past studies showing GLP-1RAs improve kidney function,^6^^,^^45^ our three drugs also partly brought back BUN, UA, and CRE levels in UUO and aged mice. However, in the UUO model, interpretation of these results should consider the potential impact of compensatory contralateral kidney function on overall renal functional recovery. Therefore, these physiological parameters can be regarded as supportive evidence for renal functional restoration, and the renoprotective effects of the drugs should be comprehensively evaluated together with histopathological and molecular analyses. Furthermore, all physiological measurements in this study were performed only at the endpoint; serial monitoring during the treatment period would help further elucidate the temporal dynamics of these effects.

Semaglutide, tirzepatide, and retatrutide exhibited pronounced anti-inflammatory and anti-fibrotic effects in the UUO model and significant anti-fibrotic activity in the aging model. At the doses tested, tirzepatide and retatrutide showed greater efficacy than semaglutide, with retatrutide being the most effective in these models, a finding consistent with that reported in diabetic nephropathy.^13^ However, the establishment of this conclusion also requires consideration of the differences in receptor equivalence and the assessment of drug efficacy. Although equipotent activation of GLP-1, GIP, and GCG receptors cannot be assured at the dose used in this study. However, studies show that retatrutide has the lowest potency,^46^ but the results demonstrate that its renoprotective effect is the strongest, indicating that its therapeutic action is mediated by pathway-specific modulation rather than pharmacokinetic advantages. The exact relationship between potency and efficacy still requires further investigation. Furthermore, the modulation trends of inflammatory and fibrotic markers by the three drugs were not entirely identical between the two CKD models. This divergence likely reflects intrinsic physiological and pathological differences between the two CKD models, which may differentially influence disease mechanisms and drug responsiveness.

Previous studies have demonstrated that GLP-1RAs confer reno-protection through multiple mechanisms, including modulation of ferroptosis, oxidative stress, and the cyclic AMP (cAMP)/PKA signaling pathway.^10^^,^^47^^,^^48^ Consistent with this mechanistic diversity, our findings reveal distinct pathway-specific profiles for the tested drugs across the UUO and aging mouse models. In the UUO model, semaglutide effects on renal inflammation and fibrosis were associated primarily with PI3K-AKT inhibition. This finding contrasts with a previous report showing that a GLP-1/GIP dual agonist normalizes diabetic nephropathy via activation of PI3K-AKT signaling.^49^ However, our findings are in line with studies demonstrating antifibrotic effects mediated by suppression of the PI3K-AKT pathway, such as those reported for Fufang Shenhua Tablet (SHT) in murine models of renal fibrosis.^50^ These apparent discrepancies likely reflect differences in disease context, experimental models, and pharmacological profiles, underscoring the context-dependent roles of PI3K-AKT signaling in renal pathology. Tirzepatide effects were associated with concurrent PI3K-AKT inhibition and PPAR pathway activation. Although preliminary experimental results indicate that tirzepatide most significantly regulates PPARβ and PPARγ, and their protective effects against kidney injury have been well documented,^51^^,^^52^ detection based solely on mRNA levels is still insufficient to determine which PPAR family members play a key role in the function of tirzepatide; therefore, further validation from multiple aspects is required, including protein-level confirmation using subtype-specific antibodies, is still required for this conclusion. The more pronounced therapeutic effects of retatrutide may be related to concurrent modulation of both the PI3K-AKT and NF-κB pathways. Although mechanistic studies on retatrutide are currently limited, prior reports demonstrating exacerbated renal inflammation and fibrosis in GCGR-knockout mice provide a compelling rationale for its enhanced performance in our study.^53^^,^^54^^,^^55^ These findings suggest that additional modulation of GCG-related signaling may contribute to the broader anti-inflammatory and antifibrotic effects of retatrutide. In contrast to the pathway-specific mechanisms observed in the UUO model, a more unified mechanism emerged in the aging model, in which the anti-fibrotic effects of all three agents were linked to mitigation of G_2_/M arrest. Although KEGG analysis showed that semaglutide was primarily enriched in the NOD-like receptor pathway, activation of p38 mitogen-activated protein kinase (MAPK) within this pathway positively regulates CDK1 activity, consistent with previous reports.^56^^,^^57^ Importantly, all three drugs significantly increased CDK1 and Cyclin B1 protein expression and reduced the percentage of G_2_/M-arrested cells, indicating functional convergence on alleviating G_2_/M arrest despite differences in upstream signaling. This observation is supported by previous studies demonstrating that G_2_/M phase arrest in tubular epithelial cells (TECs) promotes profibrotic factor production and accelerates renal fibrosis,^58^ whereas alleviation of G_2_/M arrest confers renoprotective effects.^59^^,^^60^ The enhanced therapeutic efficacy of tirzepatide and retatrutide is likely attributable to their more potent upregulation of CDK1, a key G_2_/M regulator. The optimal effect of retatrutide may further stem from the unique role of GCGR activation in renal function.^53^ Although all proposed mechanisms were substantiated through interventional experiments using specific pathway agonists or inhibitors. The key causal chain at the small interfering RNA (siRNA) knockdown or overexpression level is still necessary.

In summary, this comparative study demonstrates that, in the UUO and aging mouse models, tirzepatide and retatrutide exhibit superior efficacy to semaglutide in mitigating renal inflammation and fibrosis, with retatrutide showing better therapeutic effects. Nevertheless, given that our models do not fully reflect the complexity of human CKD, these findings serve as preliminary evidence and warrant further validation in more clinically relevant settings. Mechanistically, these drugs engage distinct signaling pathways in the UUO model: semaglutide effects were associated primarily with PI3K-AKT inhibition; tirzepatide effects were associated with PI3K-AKT inhibition together with PPAR pathway activation; and retatrutide effects were associated with concurrent inhibition of both PI3K-AKT and NF-κB pathways. In contrast, in the aging model, all three drugs were linked to mitigation of G_2_/M arrest. Studies have shown that three drugs exert renal protective effects in diabetic nephropathy through the same signaling pathway,^59^^,^^61^^,^^62^^,^^63^ reflecting the potential mechanistic consistency across different etiologies. However, the progression trajectories of different diseases are inevitably distinct, underscoring the necessity of etiology-specific investigations, particularly for diabetic nephropathy. Therefore, although the mechanisms observed in our non-diabetic models may be partially applicable to diabetic nephropathy, direct validation in the corresponding disease model is still required. Our findings suggest that semaglutide, tirzepatide, and retatrutide may represent promising agents for further investigation in non-diabetic renal fibrosis models, and offer insights into their model-specific and shared pathway engagements. The comparative observations from this study provide a reference for understanding the actions of these GLP-1RAs in renal fibrosis and may inform future studies on their therapeutic applications.

### Limitations of the study

Several limitations of this study should be acknowledged. First, the research was conducted exclusively in non-diabetic murine models (UUO and aging), limiting the direct translational relevance of our findings to diabetic nephropathy, the primary clinical context for incretin-based therapies. Second, the three drugs were administered at the same mass dose without establishing equipotent receptor activation, which may have influenced the comparative efficacy ranking observed. Finally, while we used pharmacological agonists and inhibitors to validate pathway involvement, genetic loss- and gain-of-function studies (e.g., siRNA knockdown or overexpression) were not performed, which would provide stronger causal evidence for the proposed mechanisms. Future studies addressing these limitations will be necessary to advance these findings toward clinical translation.

## Resource availability

### Lead contact

Requests for further information and resources should be directed to and will be fulfilled by the lead contact, Chao Gao (gaoc@scuec.edu.cn).

### Materials availability

This study did not generate new unique reagents.

### Data and code availability

All data supporting the findings of this study are available within the article and its supplemental information files and from the corresponding author upon reasonable request. The raw sequencing data generated in this study have been deposited in the NCBI Sequence Read Archive (SRA) and are publicly accessible under BioProject accession PRJNA1447704.

## Acknowledgments

This work was supported by the following grants: 10.13039/501100001809National Natural Science Foundation of China (82370680 to C.G., 31771274 to J.S., 32270364 and 31270361 to G.Y.). Fundamental research funds for the Central Universities (CZZ23001 to C.G., CZZ21004, CZZ24011 and XTZ20021 to G.Y.). It was also supported by Hubei Medical Biology International Science and Technology Cooperation Base.

## Author contributions

M.D., conceptualization, methodology, investigation, writing – original draft, writing-review and editing, data curation, and formal analysis; X.L., methodology; Y.W., methodology; J.W., methodology; B.J., methodology; C.L., writing – review and editing; W.M., writing – review and editing; Y.P., J.S., and G.Y., resources; C.G., conceptualization, formal analysis, supervision, funding acquisition, project administration, and writing – review and editing.

## Declaration of interests

The authors declare no competing interests.

## STAR★Methods

### Key resources table

**Table:** 

REAGENT or RESOURCE	SOURCE	IDENTIFIER
**Antibodies**		

Cyclin B1	ABclonal	Cat#A19037; RRID:AB_2862529
CDK1	ABclonal	Cat#A11420; RRID: AB_2861564
IL-1β	ABclonal	Cat#A20527; RRID: AB_2924837
Collagen III	ABclonal	Cat#A0817; RRID: AB_3661637
GAPDH	Servicebio	Cat#GB15004-100; RRID:AB_2943040
α-SMA	Servicebio	Cat#GB13044-50; RRID:AB_2942067
Fibronectin	Servicebio	Cat#GB112093-50; RRID:AB_3752604
NF-κB	Hua’an Biotechnology	Cat#ET1603-12; RRID:AB_3069668
phosphorylated (P)-NF-κB	Hua’an Biotechnology	Cat#HA723223;RRID:AB_3752603
PPAR	Hua’an Biotechnology	Cat#HA500569;RRID:AB_3752608
PI3K	Cell Signaling	Cat#4257T; RRID:AB_659889
P-PI3K	Cell Signaling	Cat#17366T; RRID:AB_2895293
AKT	Cell Signaling	Cat#4685T; RRID:AB_2225340
P-AKT	Cell Signaling	Cat#4060T; RRID:AB_2315049
HRP Goat Anti Mouse IgG (H + L)	Servicebio	Cat#GB23301; RRID:AB_2904020
HRP Goat Anti Rabbit IgG (H + L)	Servicebio	Cat#GB23303; RRID:AB_2811189

**Biological samples**		

Mouse kidney samples	This paper	N/A
Mouse skeletal muscle sample	This paper	N/A

**Chemicals, peptides, and recombinant proteins**		

Semaglutide	MedChemExpress	CAS: 910463-68-2
Tirzepatide	MedChemExpress	CAS: 2023788-19-2
Retatrutide	MedChemExpress	CAS:2381089-83-2
NF-κB agonist Resiquimod (R-848)	Yeasen Biotechnology	CAS: 144875-48-9
PPAR inhibitor GW9662	Yeasen Biotechnology	CAS: 22978-25-2
PI3K-AKT agonist 740 Y-P	Yeasen Biotechnology	CAS: 1236188-16-1
The G_2_/M phase inhibitor RO-3306	Beyotime Biotechnology	CAS: 872573-93-8
Recombinant Human TGF-beta 1	Solarbio	CAS: P00121
Cisplatin (CDDP)	Beyotime	CAS: ST1164-50 mg

**Critical commercial assays**		

Senescence β-Galactosidase Staining Kit	Beyotime	CAS: C0602
Uric acid (UA) Test Kit	Nanjing Jiancheng	CAS: C012-2-1
Urea Assay Kit	Nanjing Jiancheng	CAS: C013-2-1
Creatinine (Cr) Assay kit (sarcosine oxidase)	Nanjing Jiancheng	CAS: C011-2-1
Mouse FN ELISA Kit	Zcibio	CAS: ZC-38792
Mouse IL-6 ELISA Kit	ABclonal	CAS: RK00008
Mouse IL-1 beta High Sensitivity ELISA Kit	ABclonal	CAS: RK04878
MolPure® TRIeasy Plus Total RNA Kit	Yeasen	CAS:19211ES60
Hieff® qPCR SYBR Green Master Mix (High Rox Plus)	Yeasen	CAS:11203ES08
ABScript II cDNA First-Strand Synthesis Kit	ABclonal	CAS: RK20400

**Deposited data**		

Raw RNA sequencing data file	NCBI Sequence Read Archive (SRA)	BioProject: PRJNA1447704

**Experimental models: Cell lines**		

The human renal proximal tubular (HK-2) cell	Nanjing Cobioer Biotechnology	CAS: CBP60447

**Experimental models: Organisms/strains**		

Mouse: C57BL/6J	Liaoning Changsheng Biotechnology Co., Ltd	SYXK(E)2021-0089

**Oligonucleotides**		

Primers for all, see Table S1	This paper	N/A

**Software and algorithms**		

ImageJ	National Institutes of Health (NIH)	https://imagej.net/ij/download/
CorelDRAW 2019 (64-Bit)	Alludo (formerly Corel Corporation)	https://www.coreldraw.com; Oshima et al.^1^
GraphPad Prism (Version 9.5)	GraphPad Software, Inc.	https://www.graphpad.com/updates; Niu et al.^4^
ModFit 5.0	Verity Software House	https://vsh.com; Bucciarelli et al.^7^

**Other**		

RNA sequencing detection	BGI Genomics and majorbio	N/A

### Experimental model and study participant details

Male C57BL/6J mice (2- and 16-month-old) mice were purchased from Liaoning Changsheng Biotechnology Co., Ltd. (Liaoning, China; license: SYXK(E)2021-0089). All animals were housed in a specific pathogen-free (SPF) facility under controlled conditions (22 ± 2°C, 55 ± 10% humidity, 12-h light/dark cycle) with free access to standard chow and water. All animal care and handling procedures were approved by the Institutional Animal Care and Use Committee of the South-Central Minzu University. The ethics approval number is 2023-scuec-025. The human renal proximal tubular (HK-2) cell line was acquired from Nanjing Cobioer Biotechnology Co., Ltd. (CAS: CBP60447).

### Method details

#### Animal model establishment and experimental design

The mice were randomly assigned to the following groups: wild-type control (WT, 2-month-old), UUO/Aging model (UUO/Aging), Semaglutide-treated UUO/Aging model (Semaglutide), Tirzepatide-treated UUO/Aging model (Tirzepatide), and Retatrutide-treated UUO/Aging model (Retatrutide). Drug-treated UUO model mice received drug treatment on day 8 post-surgery. Aged mice were treated directly at 16 months of age. The drug was dissolved in physiological saline and administered daily for 14 days (120 μg/kg/day).^64^ Control mice received an equal volume of saline. Body weight, food intake, and water consumption were monitored throughout the study. In designated inhibitor/agonist cohorts, the following compounds were co-administered: NF-κB agonist R-848 (3 mg/kg), PI3K-AKT agonist 740 Y-P (10 mg/kg), PPAR inhibitor GW9662 (1 mg/kg), and G_2_/M phase inhibitor RO-3306 (4 mg/kg).

#### Cell cultures and experimental protocol

HK-2 cells were cultured in DMEM/F12 medium (G4612-500 ML, Servicebio, China) supplemented with 10% fetal bovine serum (P40-37500, PAN-Biosolutions, Germany) and maintained at 37C in a humidified atmosphere of 5% CO_2_. In the fibrosis cell model, HK-2 cells were switched to serum-free medium after reaching 30% confluency and treated with TGF-β (10 ng/mL) (P00121, Solarbio, China) for 48h until approximately 80% confluency was achieved. Treatment groups were then exposed to Semaglutide, Tirzepatide, or Retatrutide (15 μg/mL) for an additional 48h. For the senescence cell model, HK-2 cells were cultured in complete medium until 50% confluency, followed by treatment with cisplatin (6 μmol/L) (CDDP; ST1164-50 mg, Beyotime, China) for 24h to induce cellular senescence. Treatment groups were administered the same concentration of the aforementioned drugs (15 μg/mL) for 48h, Additionally, the inhibitor subgroup was co-treated with RO-3306 (5 μM).

#### RNA sequencing

Total RNA was extracted from renal tissues using TRIzol® Reagent (Invitrogen). mRNA was subsequently isolated via Oligo-dT bead-based purification on a magnetic stand (Invitrogen). Sequencing libraries were prepared using the TruSeq RNA Sample Prep Kit (Illumina). Following quantification with TB5380 Picogreen (Invitrogen), bridge amplification was performed using the HiSeq 4000 PE Cluster Kit (Illumina). Paired-end sequencing was finally carried out on a HiSeq platform with the HiSeq 4000 SBS Kit (300 cycles, Illumina). After quality control of the raw sequencing data, reads were aligned to the reference genome using Hisat2, and gene expression levels (in FPKM) were quantified with RSEM. Differential expression analysis was conducted based on gene read counts using edgeR, with significant differentially expressed genes (DEGs) defined as those with a false discovery rate (FDR) < 0.05 and |log_2_FoldChange| ≥ 1. The identified DEGs were further subjected to KEGG pathway enrichment analysis and assessed for functional enrichment trends via Gene Set Enrichment Analysis (GSEA).

#### β-Galactosidase staining

HK-2 cells were seeded uniformly in 6-well plates and assigned to the following experimental groups: WT, CDDP-treated, Semaglutide-treated, Tirzepatide-treated, Retatrutide-treated, and inhibitor co-treatment groups. After 24h, cellular senescence was assessed using a Senescence β-Galactosidase Staining Kit (C0602,Beyotime, China) according to the manufacturer’s instructions, and representative photomicrographs were acquired. The percentage of senescent cells (stained blue) was quantified using ImageJ software, calculated as (area of blue staining/total area) × 100%. All experiments were performed in triplicate.

#### Flow cytometry analysis of cell cycle

Following collection, HK-2 cells were pelleted by centrifugation and the supernatant was discarded. The cell pellet was washed with ice-cold PBS and subsequently fixed in pre-chilled 70% ethanol for 8h at 4C. After fixation, cells were centrifuged and resuspended in propidium iodide (PI) staining solution, followed by incubation at 37C for 30min in the dark. Cell cycle distribution was analyzed using a BD Accuri C6 Plus flow cytometer (USA), and data were processed with ModFit 5.0 software. All experiments were conducted in triplicate.

#### Western blot (WB) analysis

Proteins from cells and tissues were extracted using RIPA lysis buffer (G2002-100 ML, Servicebio, China) supplemented with 1% phosphatase inhibitor (G2008-1 ML, Servicebio, China). Protein concentration was determined with a BCA assay kit (P0010S, Beyotime, China). The proteins were separated by SDS-PAGE. Immunoreactive bands were visualized using the Bio-Rad ChemiDoc system and quantified with ImageJ software. The following primary antibodies were used: rabbit anti-Cyclin B1 (A19037), rabbit anti-CDK1 (A11420), rabbit anti-IL-1β (A20527), and rabbit anti-Collagen III (A0817) were purchased from ABclonal; rabbit anti-GAPDH (GB15004-100), rabbit anti-α-smooth muscle actin (α-SMA, GB13044-50), and rabbit anti-Fibronectin (GB112093-50) were obtained from Servicebio; rabbit anti-NF-κB (ET1603-12), rabbit anti-phosphorylated (P)-NF-κB (HA723223), and rabbit anti-PPAR (HA500569) were purchased from Hua’an Biotechnology; rabbit anti-PI3K (4257T), rabbit anti-P-PI3K (17366T), rabbit anti-AKT (4685T), and rabbit anti-P-AKT (4060T) were acquired from Cell Signaling Technology. All WB experiments were independently repeated three times, and n indicates the number of independent biological replicates.

#### Assessment of renal function

Urine and orbital venous blood samples were collected from the mice. The blood samples were centrifuged at 4°C and 4000 rpm for 15min to isolate serum. The levels of uric acid (UA, C012-2-1), Blood urea nitrogen (BUN, C013-2-1), and serum creatinine (CRE, C011-2-1) in each group were measured using commercial assay kits (Nanjing Jiancheng, Nanjing, China). All experiments were performed in triplicate.

#### Enzyme-linked immunosorbent assay (ELISA)

Serum samples were collected from mice in each group. The expressions of Fibronectin (ZC-38792, Zcibio, China), IL-6 (RK00008, ABclonal, China), and IL-1β (RK04878, ABclonal, China) were detected using ELISA kits.

#### Measurement of urine ion concentrations

Urine samples were diluted 100-fold with deionized water, and urinary Na^+^ and K^+^ concentrations were subsequently determined using a flame spectrophotometer (YOKE INSTRUMENT, Shanghai, China).

#### Measurement of blood pressure in mice

Following the treatment period, systolic and diastolic blood pressure were determined in all groups using a tail-cuff system (BP-2010A, Softron, Beijing, China). Briefly, conscious mice were gently secured in restrainers and placed on a warm pad to maintain vasodilation. The tail cuff was positioned at the base of the tail, and blood pressure values were recorded after the mice had acclimated to the apparatus.

#### Histopathological analysis

Upon completion of the treatment, mice from each group were anesthetized and perfused transcardially with phosphate-buffered saline (PBS), followed by 4% paraformaldehyde (PFA; G1101-500 ML, Servicebio, China) for kidney fixation. The right kidney was then dissected, post-fixed by immersion in 4% PFA for 8h, and processed for paraffin embedding. Tissue sections were prepared and subsequently stained with H&E, Masson’s trichrome, and for immunohistochemistry (IHC) and immunofluorescence (IF). Stained sections were then evaluated and statistically analyzed. For H&E staining, renal injury was semi-quantitatively scored based on the severity of glomerular damage and renal cortical tubular dilation/wrinkling. Scores were assigned on a scale of 1–4, with higher scores indicating more severe injury. For Masson’s trichrome, IHC, and IF staining, positively stained areas were quantified using ImageJ software. The percentage of positive area was calculated as: (Positive area/Total area) × 100%.

#### Quantitative polymerase chain reaction (qPCR)

Total RNA was isolated from kidney tissues and HK-2 cells with a commercial kit (19211ES60, Yeasen, China) and reverse-transcribed into cDNA (RK20400, ABclonal, China). Subsequent qPCR amplification (11203ES08, Yeasen, China) was carried out with an initial denaturation step (95°C for 3min), followed by 40 cycles of 95°C for 5s and 60°C for 30s. The *GAPDH* gene served as an internal control for data normalization. The sequences of all primers used are provided in Table S1. The experiment was repeated three times independently.

### Quantification and statistical analysis

GraphPad Prism software (Version 9.5) was used for statistical analysis of the data in this study, and CorelDRAW 2019 software (64-Bit) was employed for figure composition. All data were expressed as mean ± standard error of the mean (SEM). In all experiments, *n* indicates the number of independent biological replicates: for *in vivo* studies, *n* = number of mice per group; for *in vitro* studies, *n* = number of independently cultured samples. One-way ANOVA followed by Tukey’s post hoc test was used for comparisons involving five or more groups; for comparisons between two specific groups, two-tailed Student’s *t* test was used. A *p*-value <0.05 was considered statistically significant.
