# Supplementary material for: Comparative effects of semaglutide tirzepatide and retatrutide on renal fibrosis in UUO and aged mice
Source: iScience. 2026 Aug 13;29(9):117174. doi: 10.1016/j.isci.2026.117174 (PMC13495082; doi:10.1016/j.isci.2026.117174)
Supplement: Supplementary file 1 [file mmc1.pdf]

iScience, Volume *29*

Supplemental information

Comparative effects of semaglutide tirzepatide and retatrutide on renal fibrosis in UUO and aged mice

Mingling Ding, Xiaoxiao Li, Yuchao Wei, Jitong Wang, Bohan Jiao, Caiyue Li, Wenbo Ma, Yongbo Peng, Jinhua Shen, Guanghui Yu, and Chao Gao

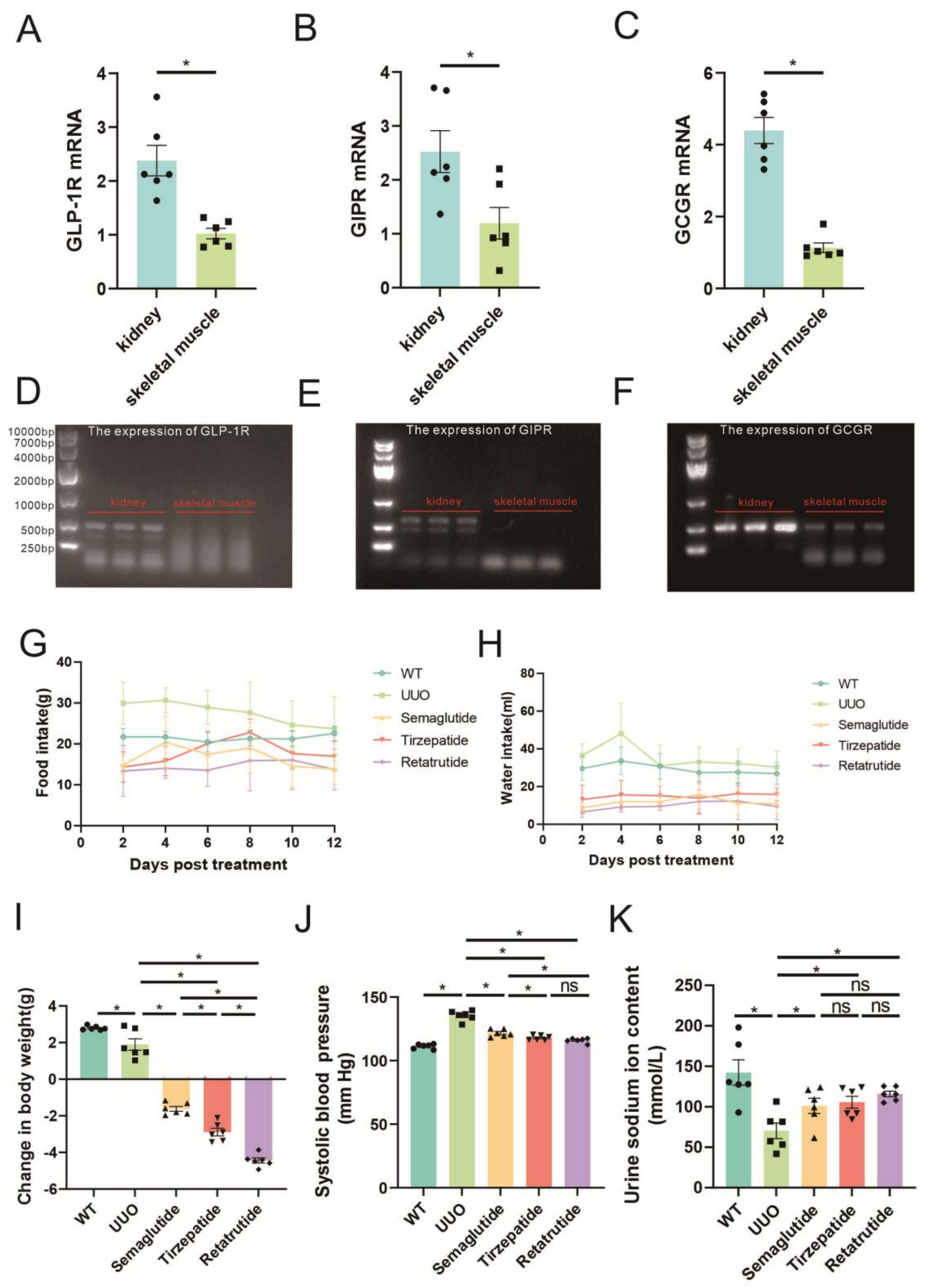

Figure S1. Semaglutide, Tirzepatide, and Retatrutide effectively alleviate renal function impairment in UUO mice. (A-C) Relative mRNA expression levels of GLP-1R, GIPR and GCGR in mouse skeletal muscle and kidney tissues (n=6). (D-F) Electrophoretic gel images of the amplified GLP-1R, GIPR and GCGR genes (n=3). (G) The changes in food intake for each group every two days

(n=6). (H) The changes in water intake for each group every two days (n=6). (I) The changes in weight of each group (n=6). (J) Systolic blood pressure (n=6). (K) Changes in urinary Na^+ concentration (n=6). Data are represented as mean ± SEM, one way ANOVA followed by Tukey's post hoc test was used for comparisons involving five groups; for comparisons between two specific groups, two tailed Student's t test was used; n: the number of independent biological replicates, *$p<0.05$.

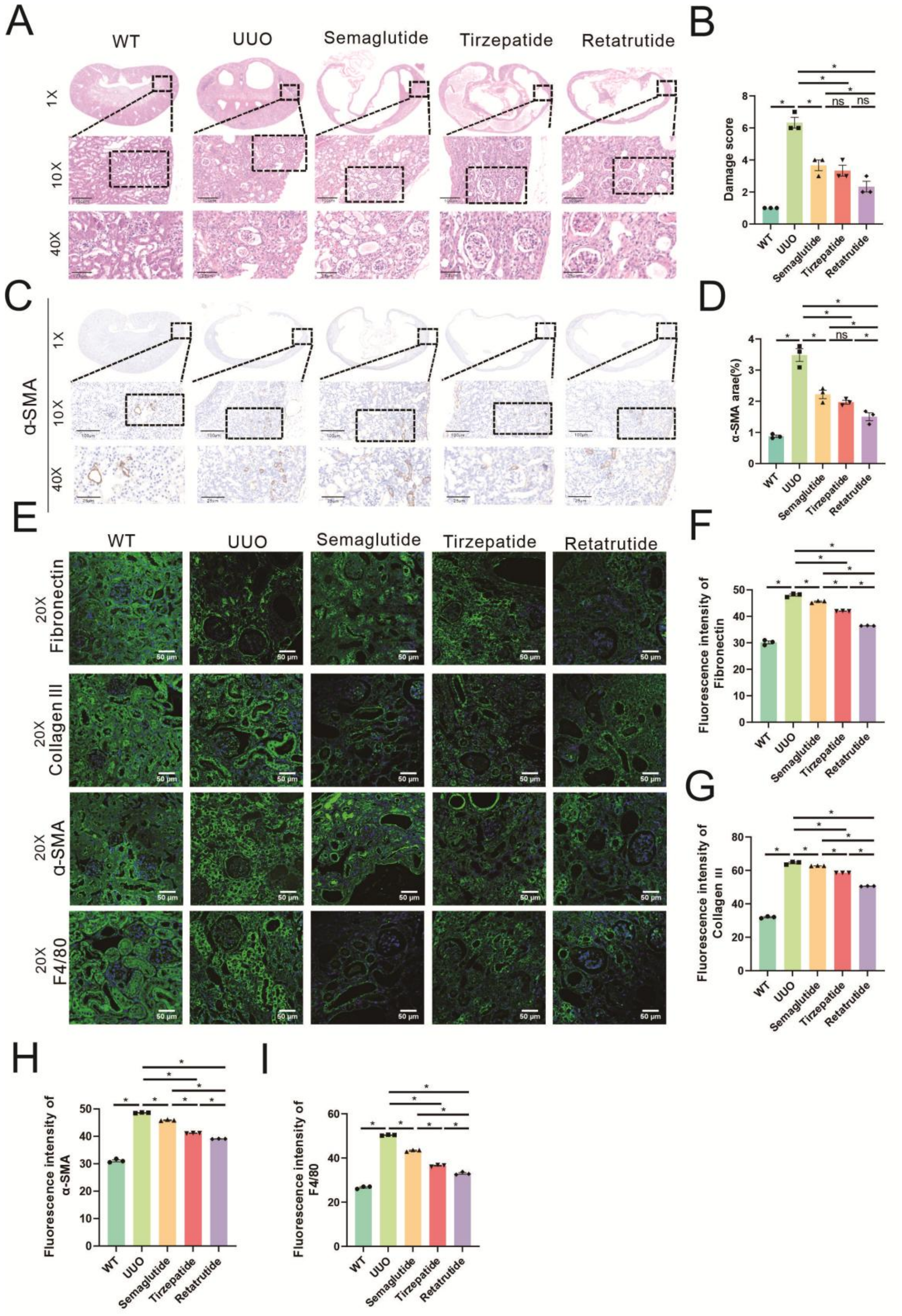

Figure S2. Semaglutide, Tirzepatide, and Retatrutide attenuate renal fibrosis and inflammation in UUO mice. (A) H&E staining of kidney tissue. Scale bars, 100 μm (upper) and 25 μm (lower). (B) Quantitative analysis of H&E staining.

(C) IHC detection of α-SMA expression. Scale bars, 100 μm (upper) and 25 μm (lower). (D) Statistical analysis of α-SMA-positive area from IHC. (E) IF detection of Fibronectin, Collagen III, α-SMA, and F4/80 expression. Scale bars, 50 μm. (F-I) Percentage of positive area for IF staining of Fibronectin, Collagen III, α-SMA, and F4/80. Data are represented as mean ± SEM, significant differences were determined by one way ANOVA followed by Tukey's post hoc test; n=3, n: the number of independent biological replicates; *p<0.05.

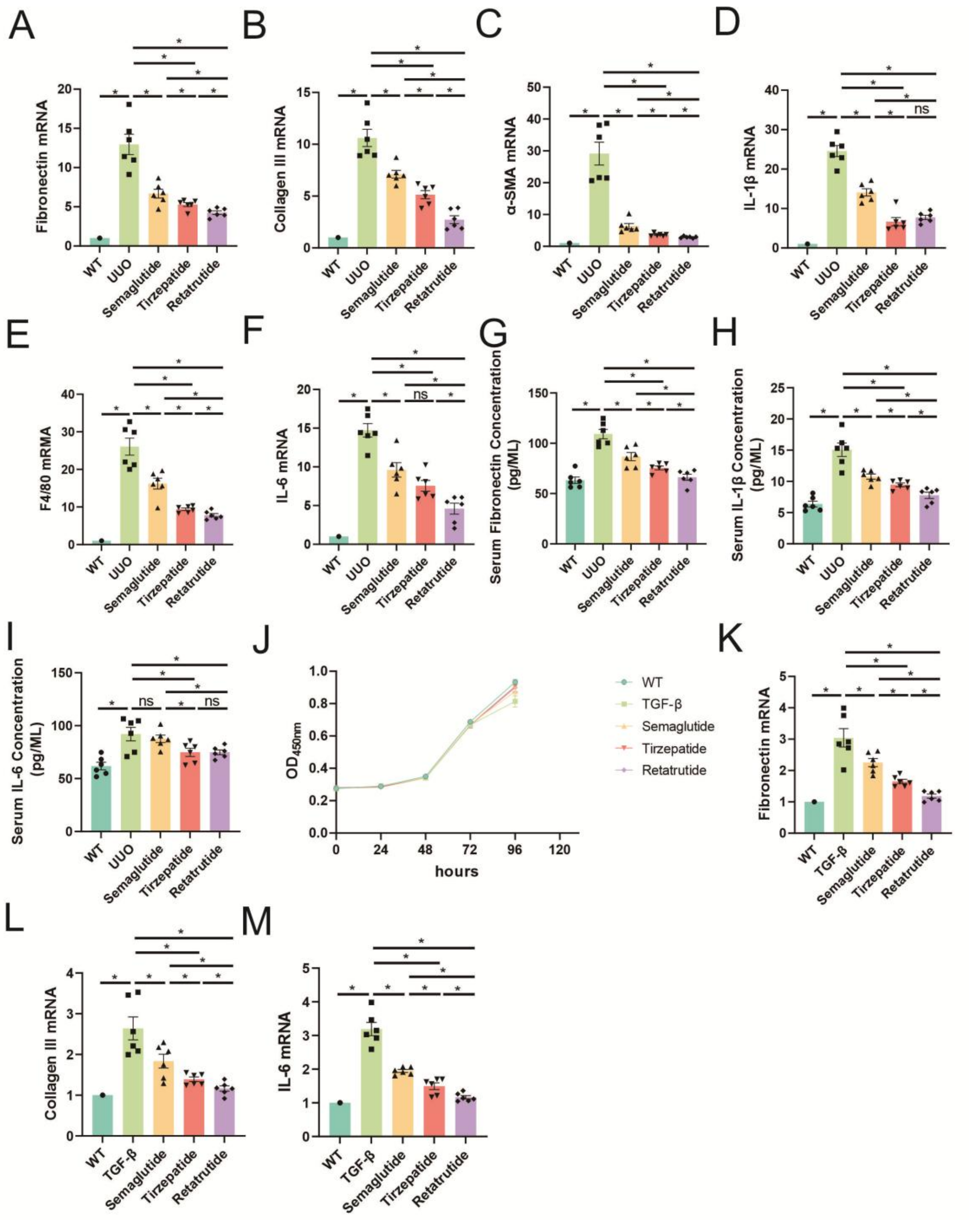

Figure S3. Drug intervention can reduce the expression of fibrosis and inflammatory factors in the kidneys of UUO mice as well as in HK-2 cells. (A-F) Relative mRNA expression levels of Fibronectin, Collagen III, α-SMA, IL-1β, F4/80, and IL-6 in mouse kidney tissues (n=6). (G-I) Serum levels of Fibronectin, IL-1β, and IL-6 detected by ELISA in each group (n=6). (J) Effects of the three drugs on HK-2 cell viability measured by CCK-8 assay (n=3). (K-M) Relative mRNA expression levels of Fibronectin, Collagen III, and IL-6 in HK-2 cells (n=6). Data are represented as mean ± SEM, significant differences were determined by one way ANOVA followed by Tukey's post hoc test; n: the number of independent biological replicates, *$p<0.05$.

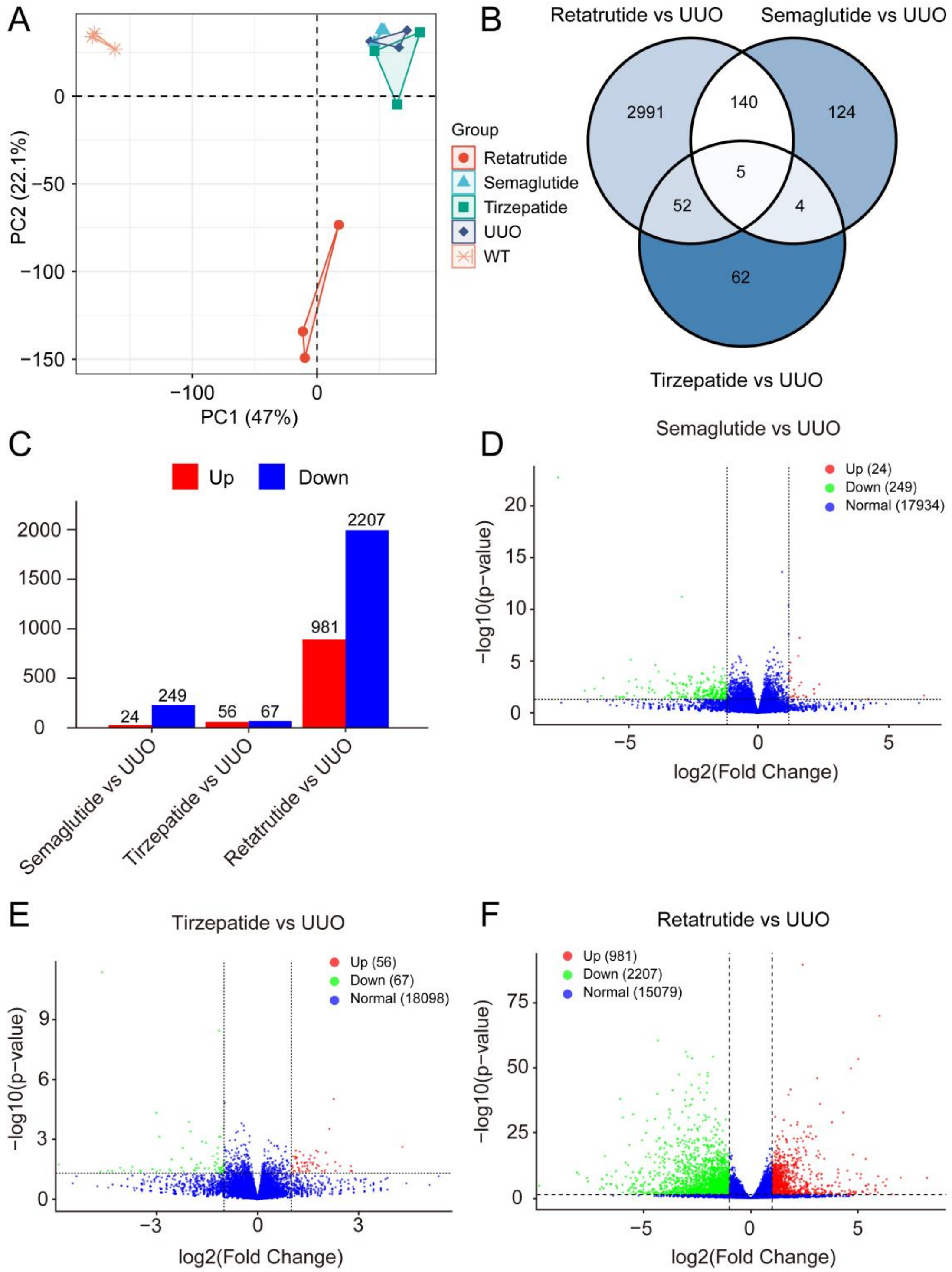

Figure S4. Analysis of transcriptome sequencing results after treatment with Semaglutide, Tirzepatide, and Retatrutide in the UUO mouse model. (A) PCA analysis diagram. (B) Venn diagram of DEGs. (C) Statistical graph of upregulated and downregulated genes in the Semaglutide, Tirzepatide, and Retatrutide groups compared to the UUO group. (D-F) Volcano plot of upregulated and downregulated genes. n=3, n: the number of independent

biological replicates.

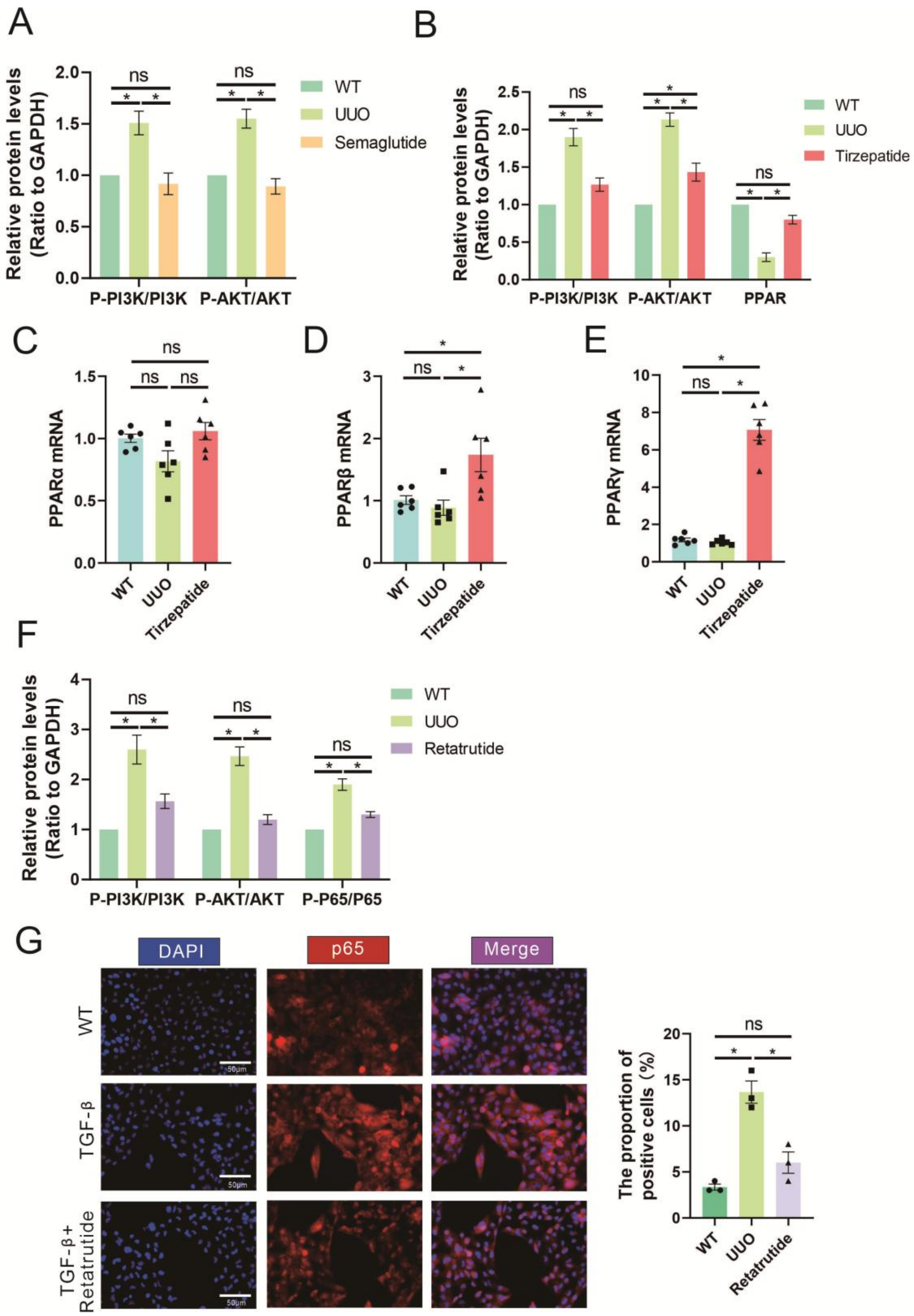

Figure S5. Statistical analysis of the effects of Semaglutide, Tirzepatide, and Retatrutide on different pathways in renal tissue. (A) Quantitative analysis of

PI3K-AKT pathway protein expression in the Semaglutide group (n=3). (B) Quantitative analysis of PI3K-AKT and PPAR pathway protein expression in the Tirzepatide group (n=3). (C-E) After treatment with Tirzepatide, the relative mRNA expression levels of PPARα, PPARβ and PPARγ in the renal tissues of mice were measured (n=6). (F) Quantitative analysis of PI3K-AKT and NF-κB pathway protein expression in the Retatrutide group (n=3). (G) The IF images of nuclear translocation of p65 in HK-2 cells and the statistics of positive cells that entered the nucleus (n=3). Scale bars, 50 μm. Data are represented as mean ± SEM, significant differences were determined by one way ANOVA followed by Tukey's post hoc test; n: the number of independent biological replicates, $*p<0.05$.

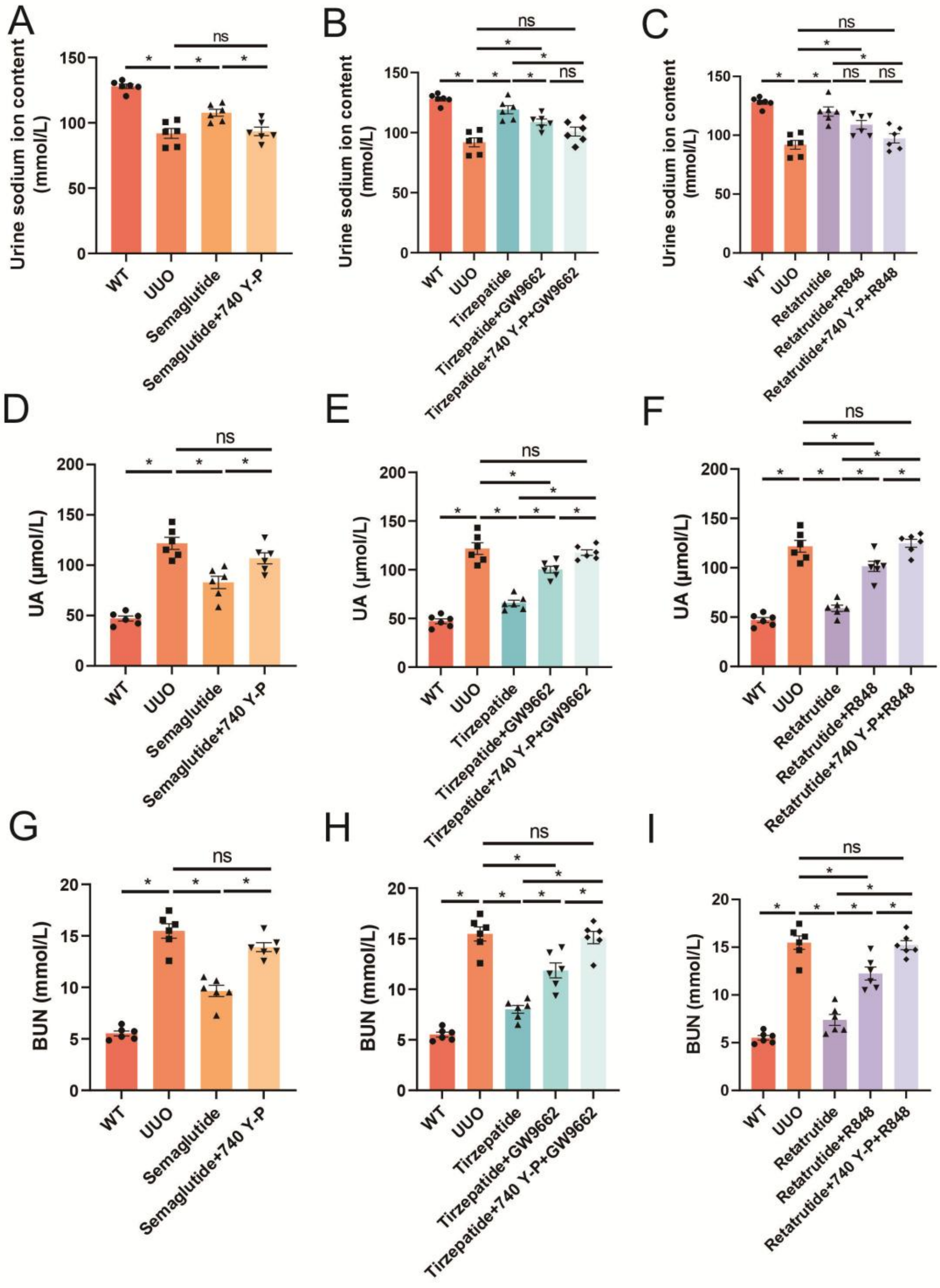

Figure S6. The regulatory effects of Semaglutide, Tirzepatide, and Retatrutide on renal injury in UUO mice are closely linked to the synergistic actions of their signaling pathways. (A-C) Urinary Na^+ levels in the Semaglutide, Tirzepatide, and Retatrutide groups before and after administration of pathway agonists/inhibitors. (D-F) Changes in UA levels in the Semaglutide, Tirzepatide, and Retatrutide groups before and after administration of pathway

agonists/inhibitors. (G-I) Changes in BUN levels in the Semaglutide, Tirzepatide, and Retatrutide groups before and after administration of pathway agonists/inhibitors. Data are represented as mean ± SEM, significant differences were determined by one way ANOVA followed by Tukey’s post hoc test; n=6, n: the number of independent biological replicates; *p<0.05.

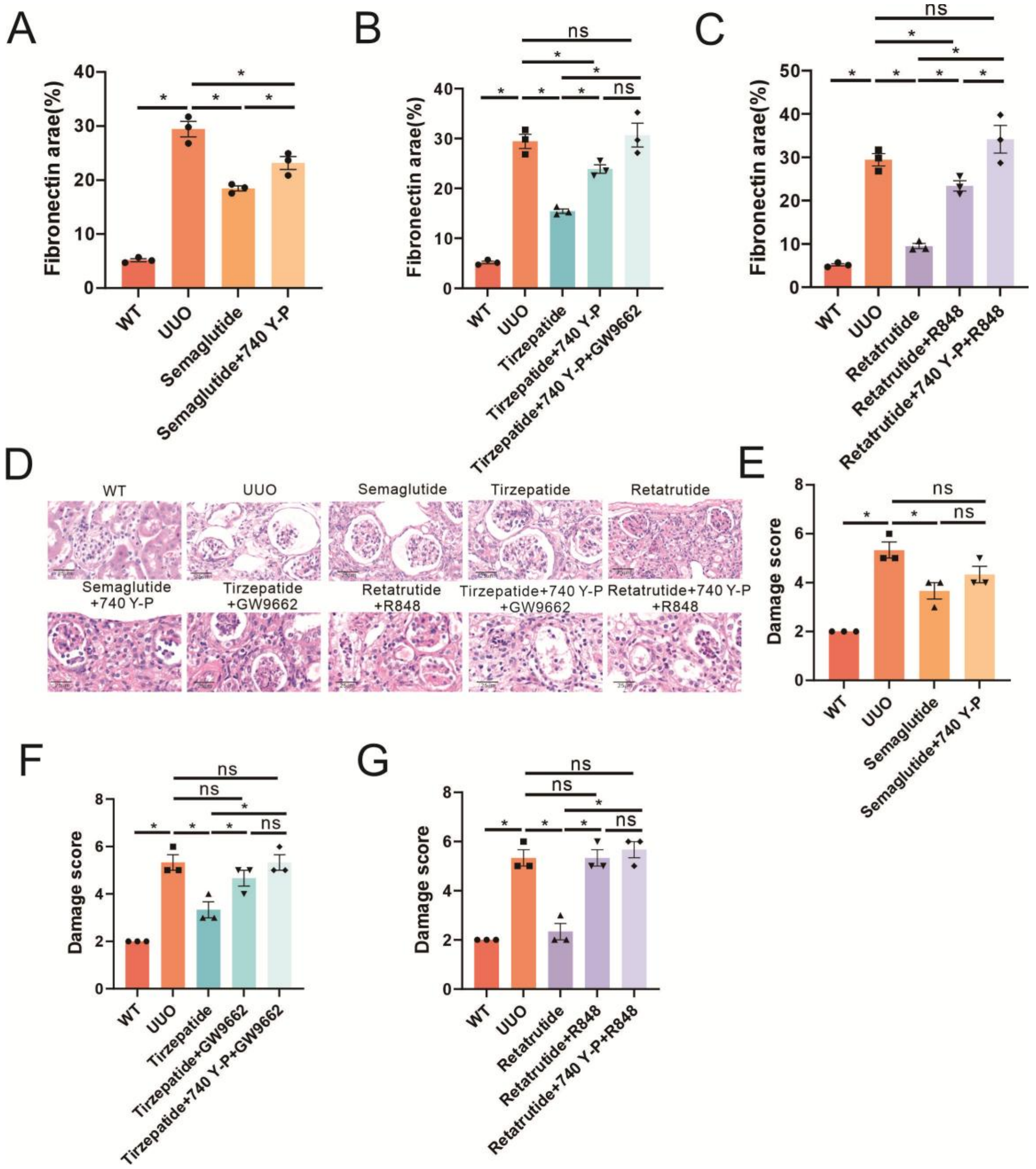

Figure S7. The regulatory effects of Semaglutide, Tirzepatide, and Retatrutide on renal injury in UUO mice are closely linked to the synergistic actions of their signaling pathways. (A-C) Quantitative analysis of the positive area in Masson's trichrome staining of kidney tissues from the Semaglutide, Tirzepatide, and

Retatrutide groups before and after treatment with pathway agonists or inhibitors. (D) Representative images of H&E staining before and after treatment with pathway agonists or inhibitors. Scale bars, 25 μm. (E-G) Quantitative analysis of H&E staining results in the Semaglutide, Tirzepatide, and Retatrutide groups. Data are represented as mean ± SEM, significant differences were determined by one way ANOVA followed by Tukey's post hoc test; n=3, n: the number of independent biological replicates; *p<0.05.

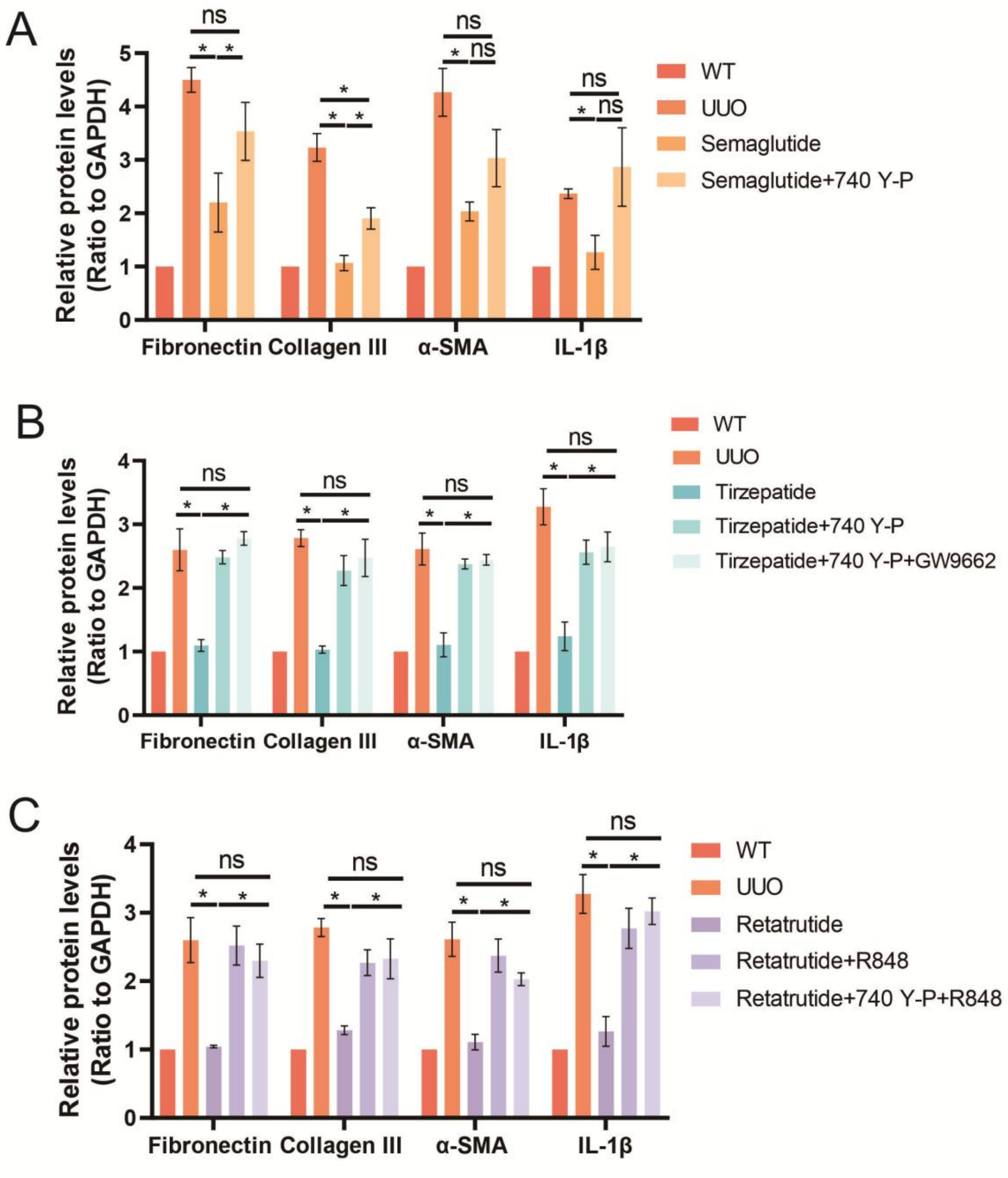

Figure S8. The regulatory effects of Semaglutide, Tirzepatide, and Retatrutide

on renal injury in UUO mice are closely linked to the synergistic actions of their signaling pathways. (A-C) Statistical analysis of WB results for renal tissue Fibronectin, Collagen III, α-SMA, and IL-1β in Semaglutide, Tirzepatide, and Retatrutide groups before and after treatment with pathway agonists or inhibitors. Data are represented as mean ± SEM, significant differences were determined by one way ANOVA followed by Tukey's post hoc test; n=3, n: the number of independent biological replicates; *p<0.05.

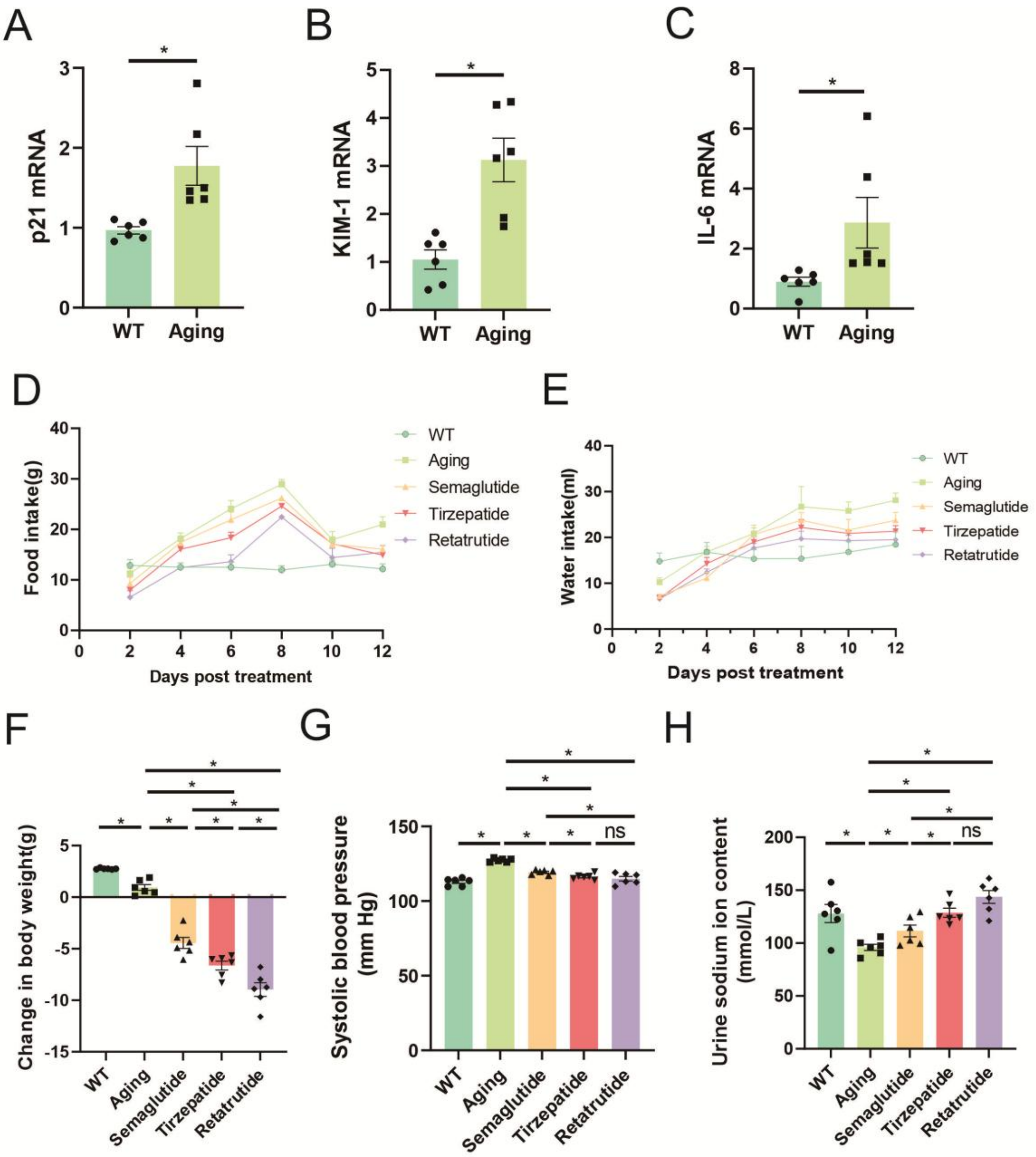

Figure S9. Semaglutide, Tirzepatide, and Retatrutide alleviate renal function impairment in aged mice. (A-C) The relative mRNA expression levels of p21, KIM-1 and IL-6 in the renal tissues of aged mice. (D) The changes in food intake

for each group every two days. (E) The changes in water intake for each group every two days. (F) Changes in body weight. (G) Systolic blood pressure (SBP). (H) Changes in urinary Na^+ concentration. Data are represented as mean ± SEM, one way ANOVA followed by Tukey's post hoc test was used for comparisons involving five groups; for comparisons between two specific groups, two tailed Student's t test was used; n=6, n: the number of independent biological replicates; *p<0.05.

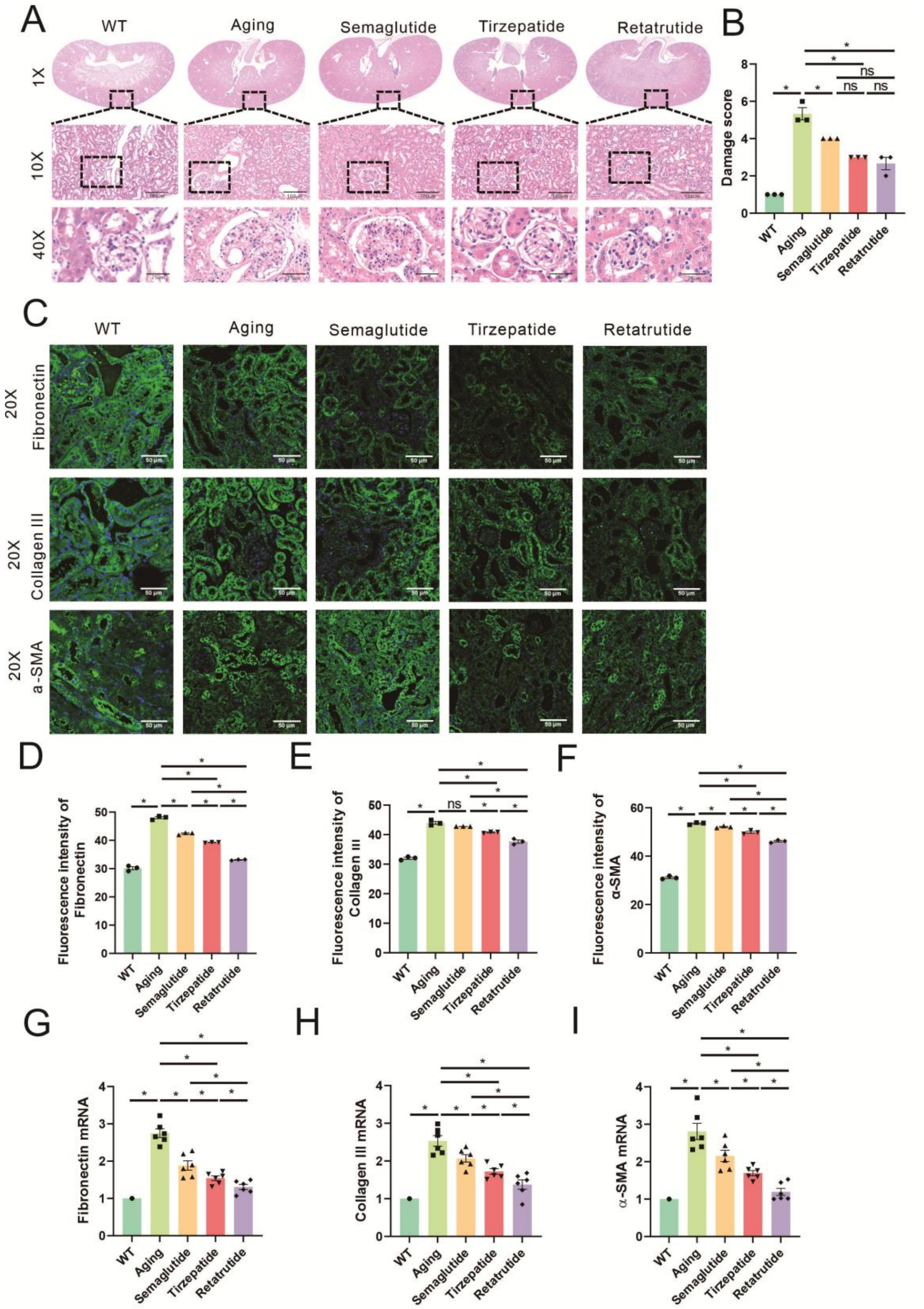

Figure S10. Semaglutide, Tirzepatide, and Retatrutide attenuate renal injury and fibrosis in aged mice. (A-B) H&E staining of kidney sections and quantitative analysis (n=3). Scale bars, 100 μm (upper) and 25 μm (lower). (C-F) IF staining for the expression of Fibronectin, Collagen III, and α-SMA, with

statistical analysis of positive area percentages (n=3). Scale bars, 50 μm. (G-I) Relative mRNA expression levels of fibrosis markers Fibronectin, Collagen III, and α-SMA as detected by qPCR (n=6). Data are represented as mean ± SEM, significant differences were determined by one way ANOVA followed by Tukey's post hoc test; n: the number of independent biological replicates, *p<0.05.

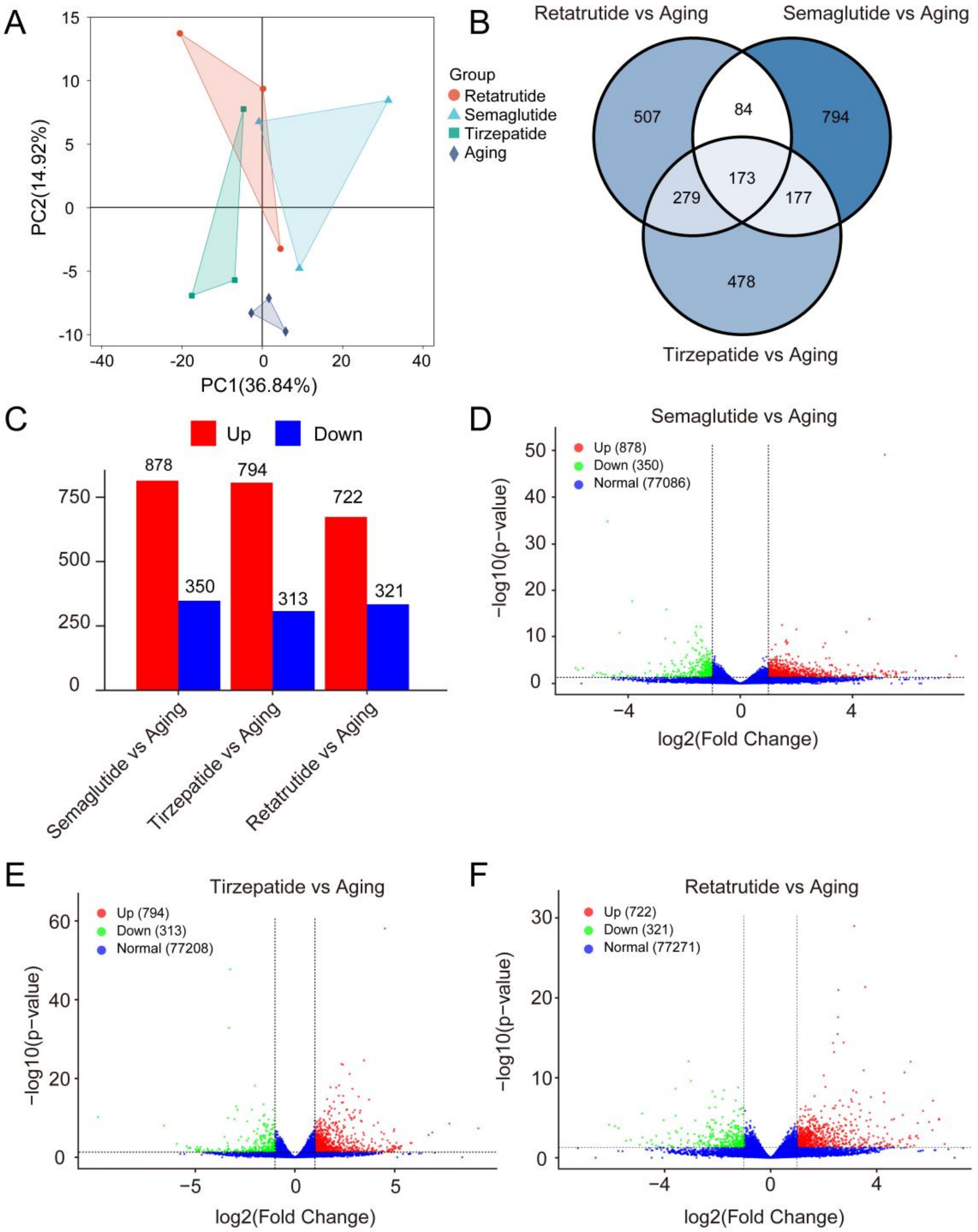

Figure S11. Analysis of transcriptome sequencing results after treatment with Semaglutide, Tirzepatide, and Retatrutide in the aging mouse model. (A) PCA

analysis diagram. (B) Venn diagram of DEGs. (C) Statistical graph of upregulated and downregulated genes in the Semaglutide, Tirzepatide, and Retatrutide groups compared to the aged group. (D-F) Volcano plot of upregulated and downregulated genes. n=3, n: the number of independent biological replicates.

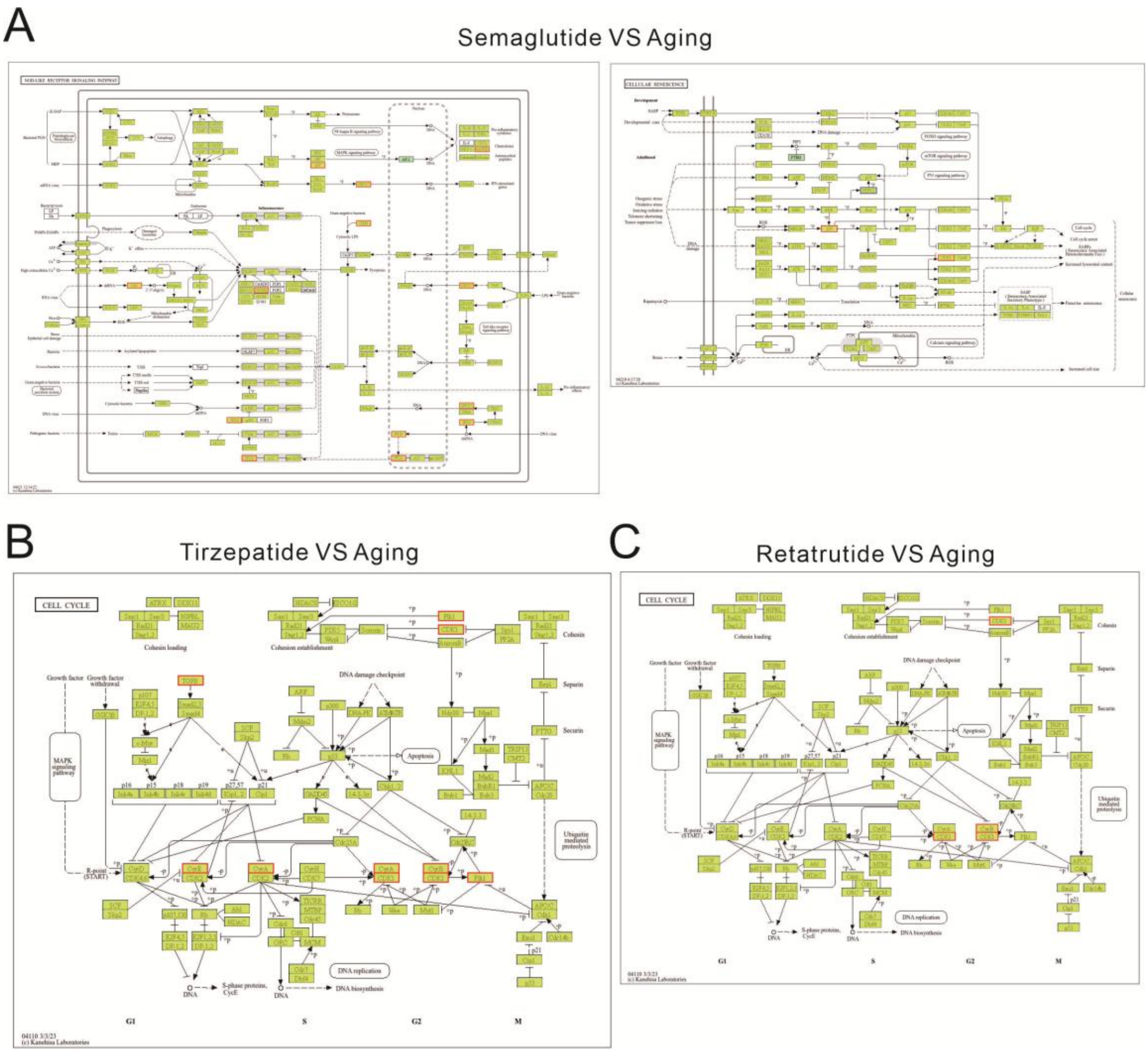

Figure S12. The signaling pathway diagrams obtained from KEGG analysis of Semaglutide, Tirzepatide and Retatrutide in aged mice. (A) Mechanism diagram of Semaglutide regulating the NOD-like receptor signaling pathway and cellular senescence in aged mice. (B) The mechanism diagram of Tirzepatide regulating cell cycle in aged mice. (C) The mechanism diagram of Retatrutide regulating cell cycle in aged mice.

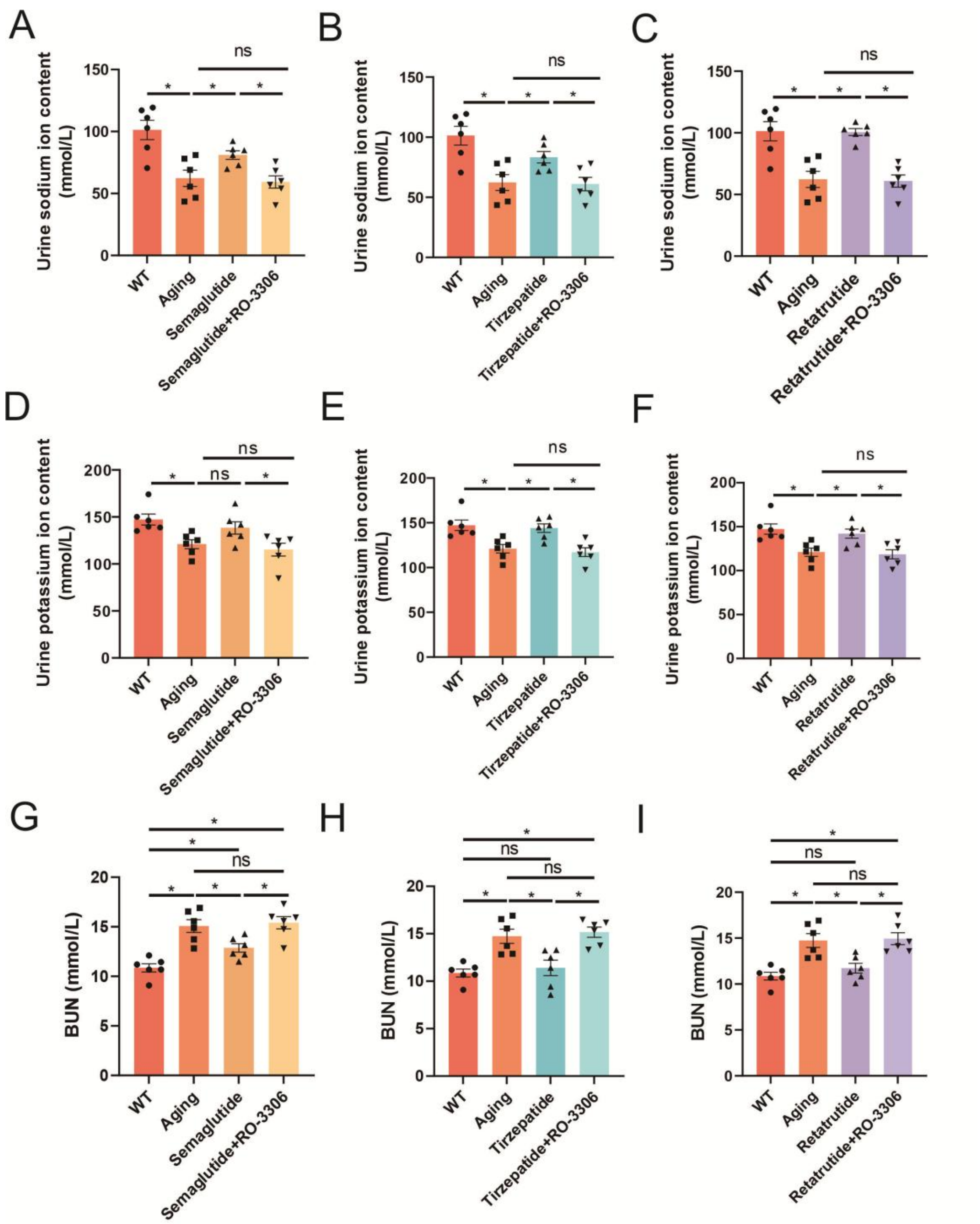

Figure S13. Semaglutide, Tirzepatide and Retatrutide alleviate renal fibrosis in the elderly by regulating the G_2/M phase. (A-F) Urinary Na^+ and K^+ levels in the Semaglutide, Tirzepatide, and Retatrutide groups before and after inhibitor treatment. (G-I) BUN levels in the Semaglutide, Tirzepatide, and Retatrutide groups before and after inhibitor treatment. Data are represented as mean ± SEM, significant differences were determined by one way ANOVA followed by Tukey's post hoc test; n=6, n: the number of independent biological replicates;

*p<0.05.

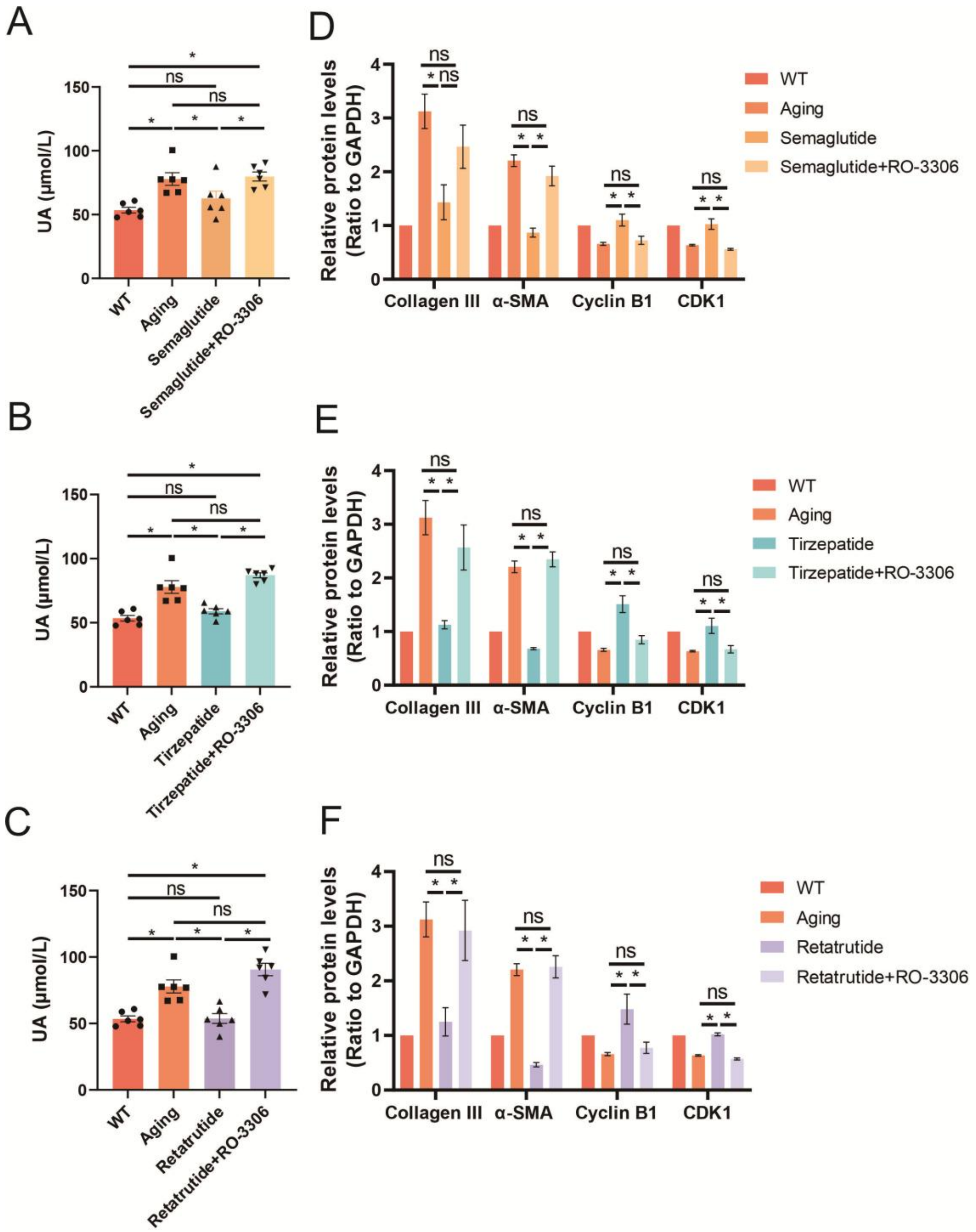

Figure S14. Semaglutide, Tirzepatide and Retatrutide alleviate renal fibrosis in the elderly by regulating the G_2/M phase. (A-C) UA levels in the Semaglutide, Tirzepatide, and Retatrutide groups before and after inhibitor treatment (n=6). (D-F) WB analysis shows the expression of Collagen III, α-SMA, Cyclin B1, and CDK1 in renal tissue from each group before and after inhibitor treatment (n=3). Data are represented as mean ± SEM, significant differences were determined

by one way ANOVA followed by Tukey's post hoc test; n: the number of independent biological replicates, *p<0.05.

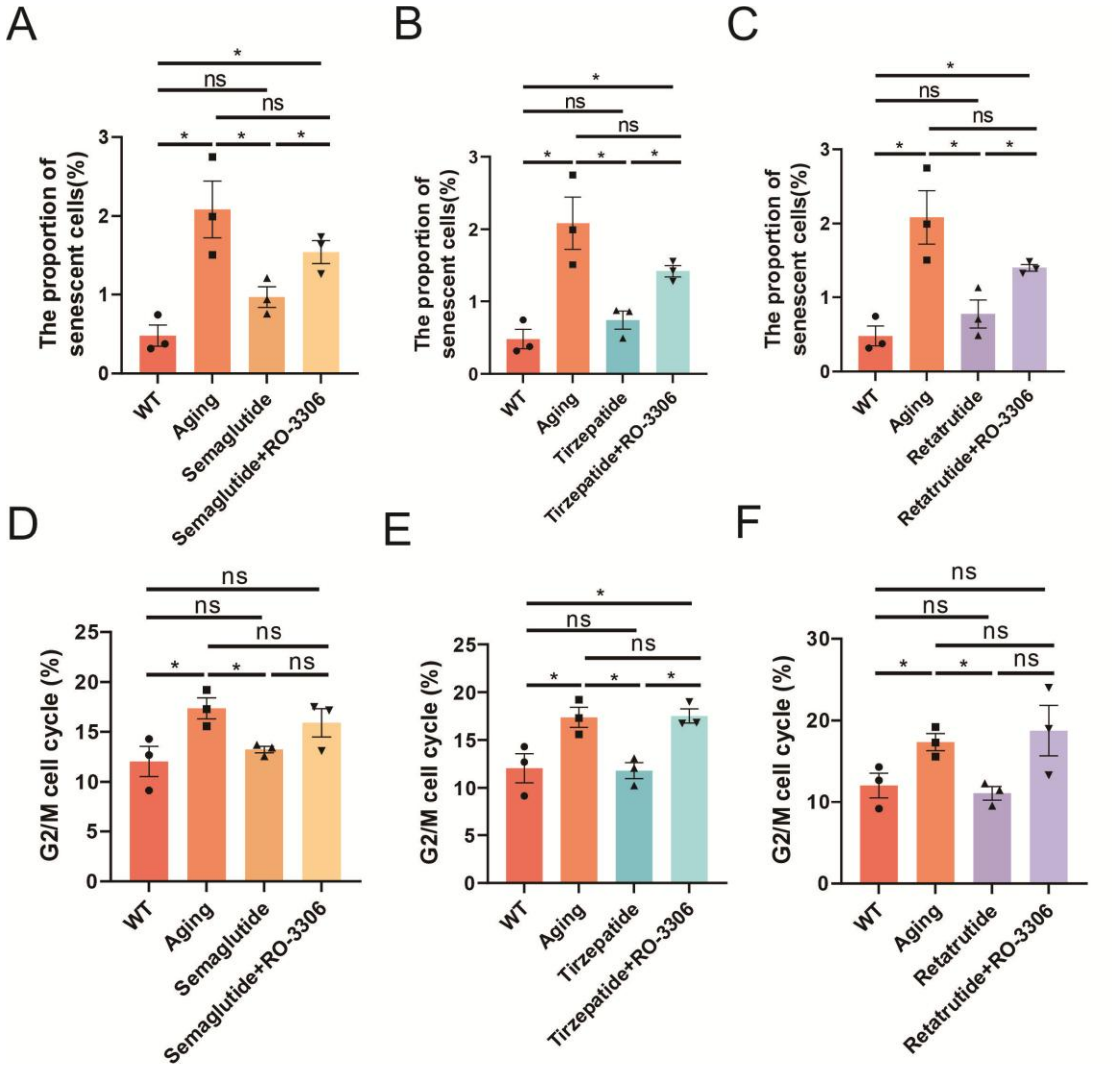

Figure S15. Semaglutide, Tirzepatide, and Retatrutide inhibit the increase in fibrotic levels induced by senescence in HK-2 cells. (A-C) Percentage of senescent cells (blue) in the Semaglutide, Tirzepatide, and Retatrutide group. (D-F) Percentage of HK-2 cells in G_2/M phase in the Semaglutide, Tirzepatide, and Retatrutide group. Data are represented as mean ± SEM, significant differences were determined by one way ANOVA followed by Tukey's post hoc test; n=3, n: the number of independent biological replicates; *p<0.05.

Table S1. Primer sequence list

Gene name	Primer	Primer sequence
Mouse Fibronectin	F	CCCTATCTCTGATACCGTTGTCC
Mouse Fibronectin	R	TGCCGCAACTACTGTGATTCGG
Mouse α-SMA	F	TGCTGACAGAGGCACCACTGAA
Mouse α-SMA	R	CAGTTGTACGTCCAGAGGCATAG
Mouse IL-1β	F	TGGACCTTCCAGGATGAGGACA
Mouse IL-1β	R	GTTCATCTCGGAGCCTGTAGTG
Mouse Collagen III	F	GACCAAAAGGTGATGCTGGACAG
Mouse Collagen III	R	CAAGACCTCGTGCTCCAGTTAG
Mouse IL-6	F	TACCACTTCACAAGTCGGAGGC
Mouse IL-6	R	CTGCAAGTGCATCATCGTTGTTC
Mouse F4/80	F	CGTGTTGTTGGTGGCACTGTGA
Mouse F4/80	R	CCACATCAGTGTTCCAGGAGAC
Mouse p21	F	TCGCTGTCTTGCACTCTGGTGT
Mouse p21	R	CCAATCTGCGCTTGGAGTGATAG
Mouse KIM-1	F	CTGGAATGGCACTGTGACATCC
Mouse KIM-1	R	GCAGATGCCAACATAGAAGCCC
Mouse GLP-1R	F	CGGAGTGTGAAGAGTCTAAGCG
Mouse GLP-1R	R	ATGGCTGAAGCGATGACCAAGG
Mouse GIPR	F	GTATGCCTGCTGGAACTACACG
Mouse GIPR	R	CACTGAGTGTGGTCTCTCCAAG
Mouse GCGR	F	CAATGCCACCACAACCTAAGCC
Mouse GCGR	R	GGCAGGAAATGTTGGCAGTGGT
Mouse PPARα	F	ACCACTACGGAGTTCACGCATG
Mouse PPARα	R	GAATCTTGCAGCTCCGATCACAC

Mouse PPARβ	F	GGACCAGAACACACGCTTCCTT
Mouse PPARβ	R	CCGACATTCCATGTTGAGGCTG
Mouse PPARγ	F	GTACTGTCGGTTTCAGAAGTGCC
Mouse PPARγ	R	ATCTCCGCCAACAGCTTCTCCT
Mouse GAPDH	F	CATCACTGCCACCCAGAAGACTG
Mouse GAPDH	R	ATGCCAGTGAGCTTCCCGTTCAG
Homo Fibronectin	F	ACAACACCGAGGTGACTGAGAC
Homo Fibronectin	R	GGACACAACGATGCTTCCTGAG
Homo Collagen III	F	TGGTCTGCAAGGAATGCCTGGA
Homo Collagen III	R	TCTTTCCCTGGGACACCATCAG
Homo IL-6	F	AGACAGCCACTCACCTCTTCAG
Homo IL-6	R	TTCTGCCAGTGCCTCTTTGCTG
Homo GAPDH	F	GTCTCCTCTGACTTCAACAGCG
Homo GAPDH	R	ACCACCCTGTTGCTGTAGCCAA

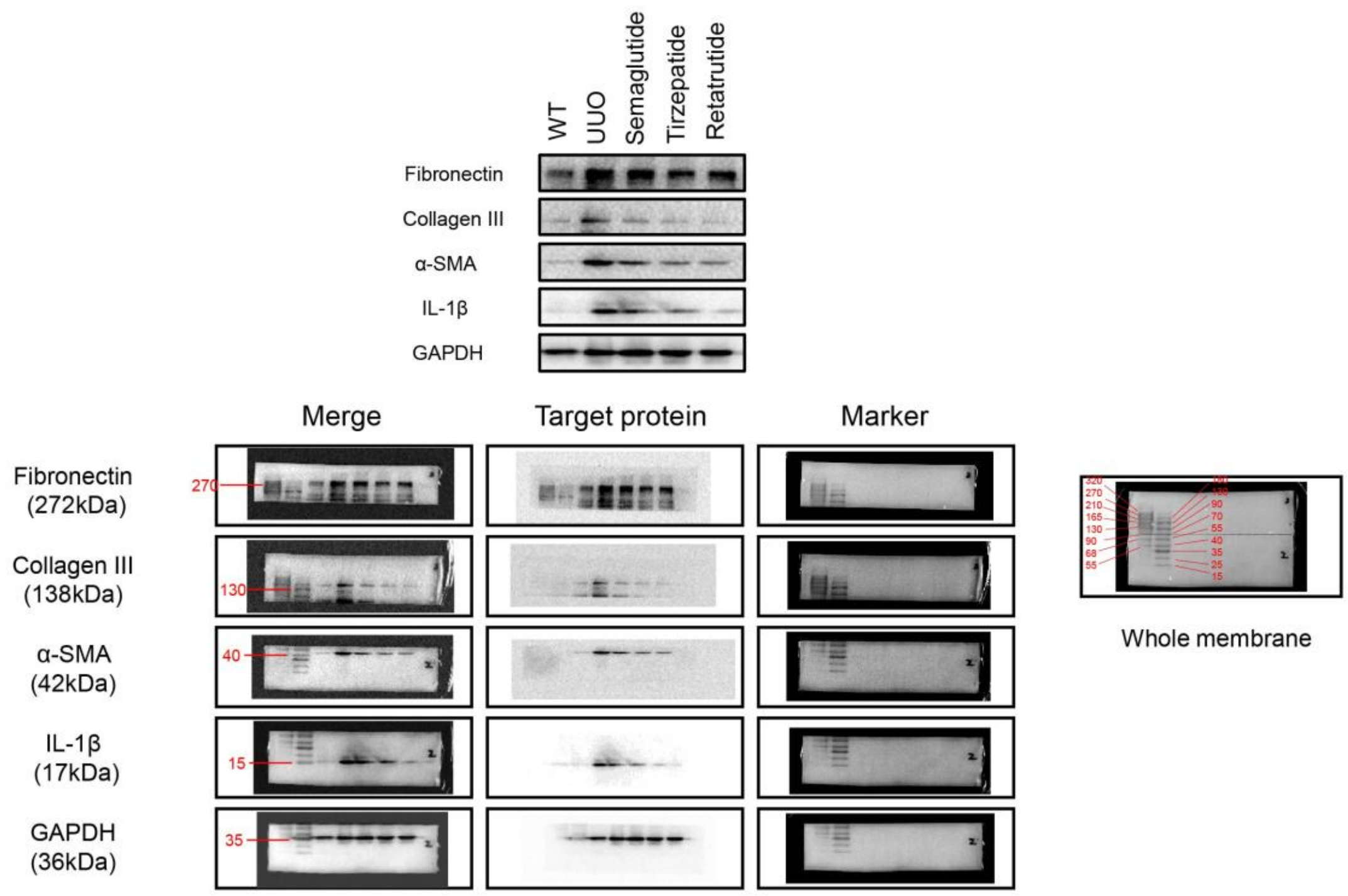

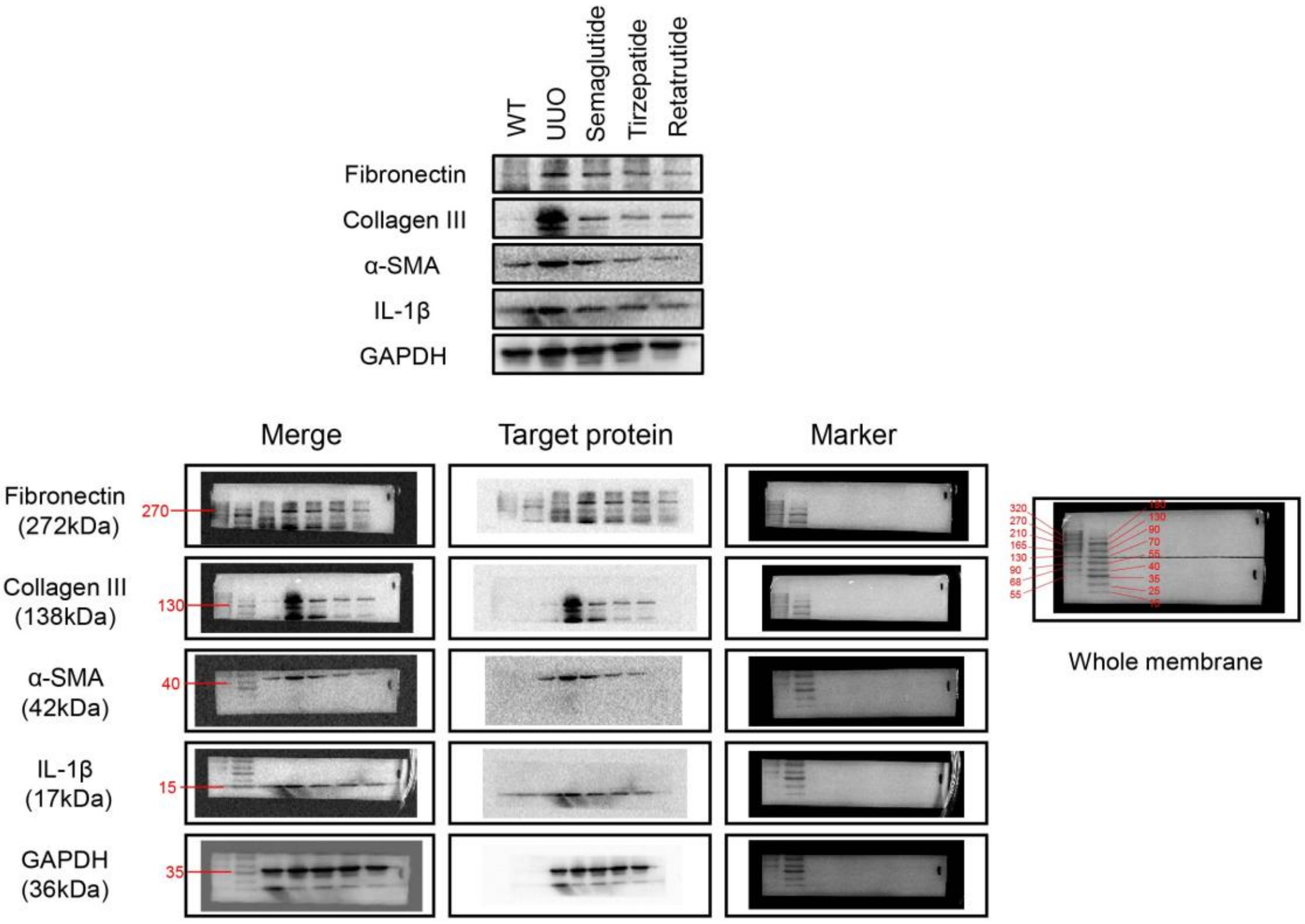

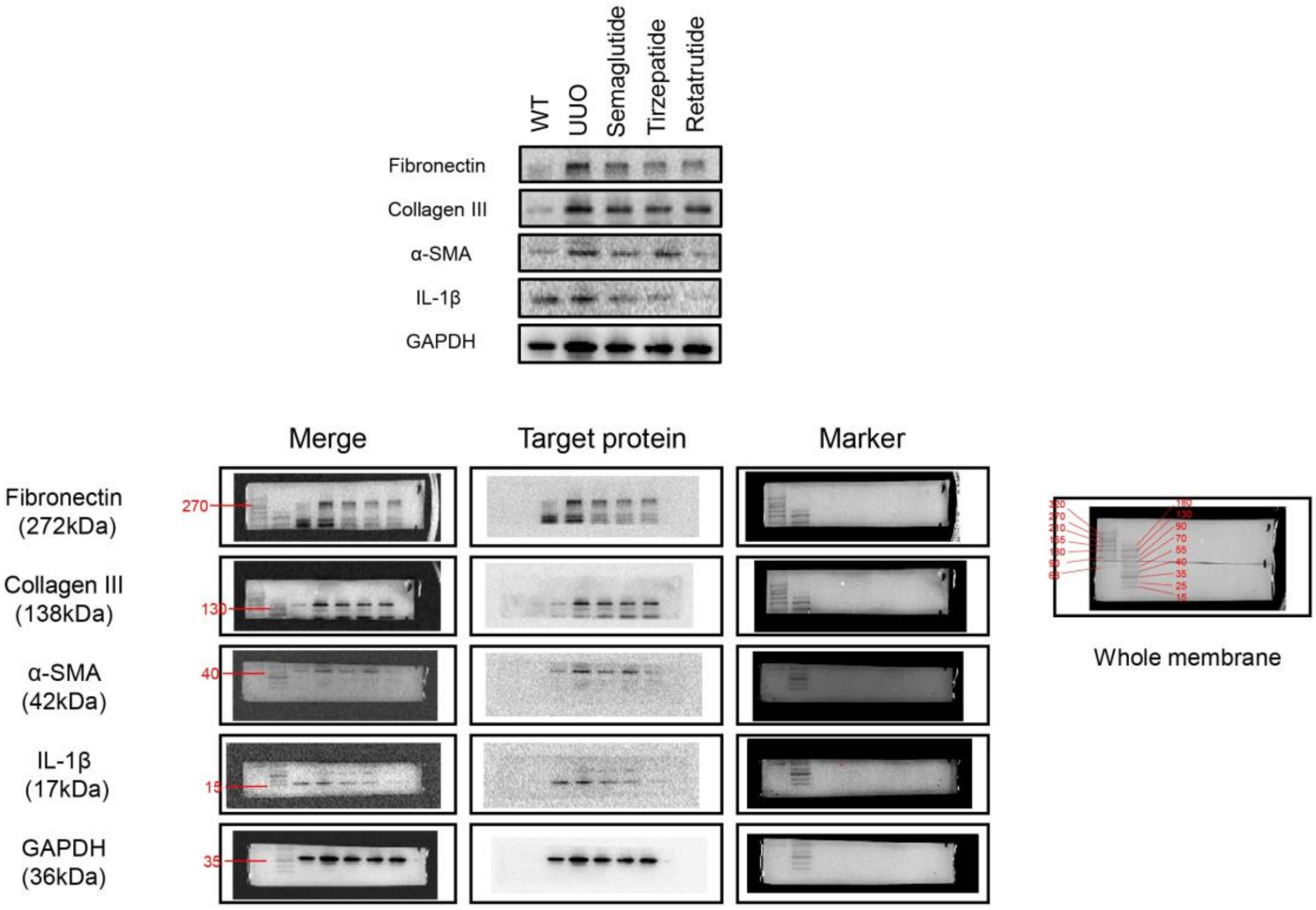

Data S1: Western blot original data for Figure 2G. (Three independent replicates were performed)

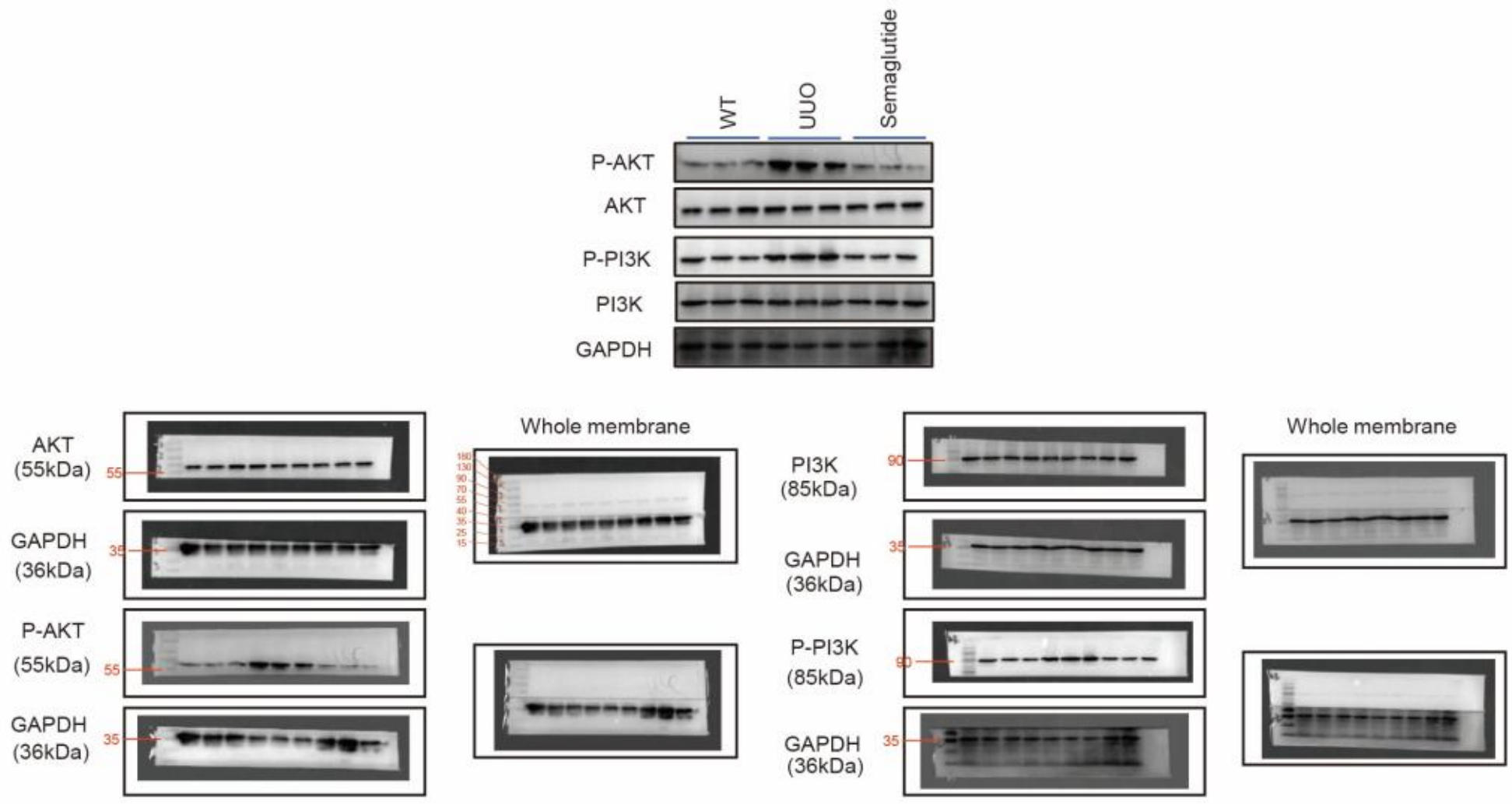

Data S2: Western blot original data for Figure 3E.

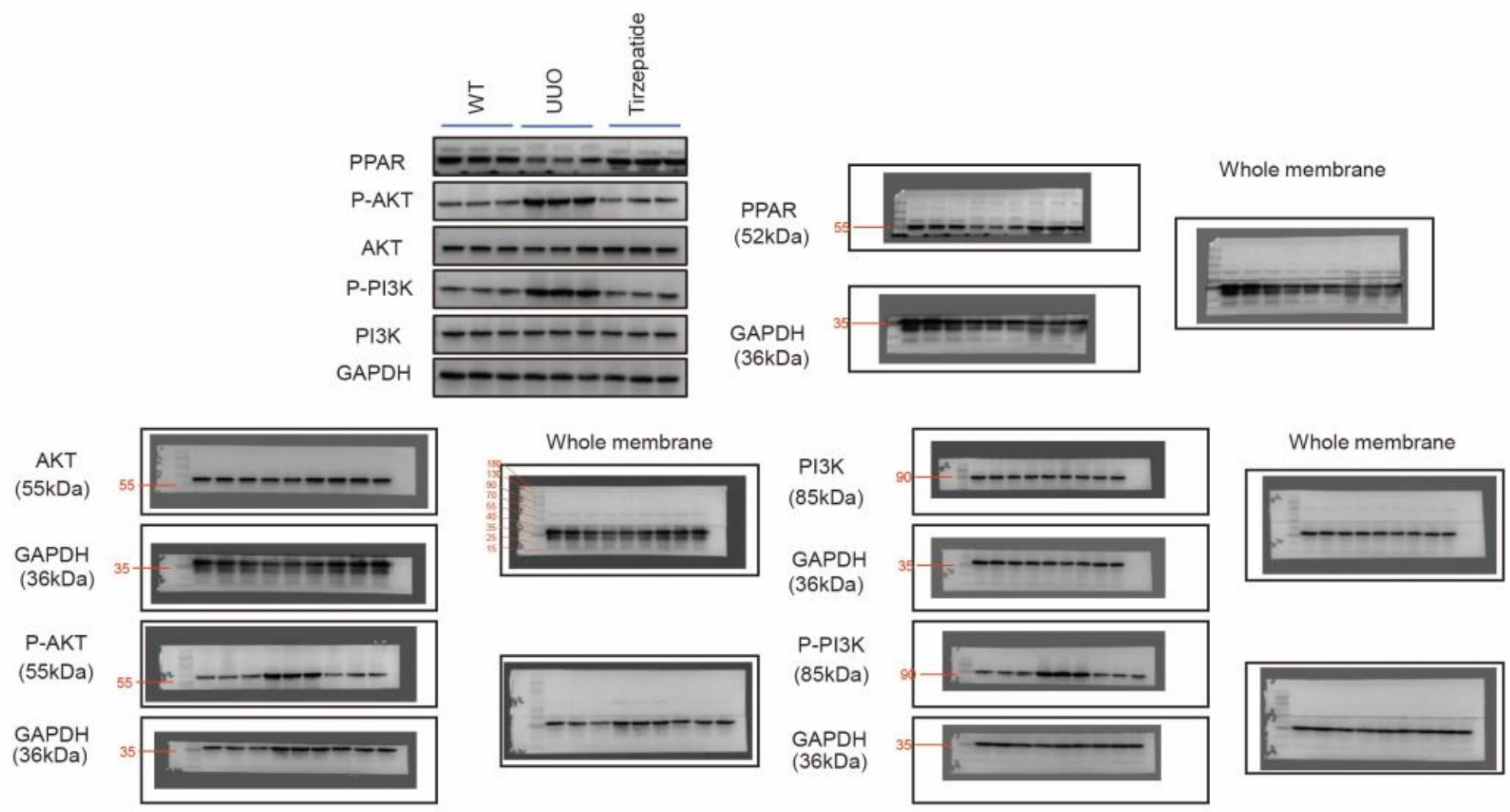

Data S3: Western blot original data for Figure 3F.

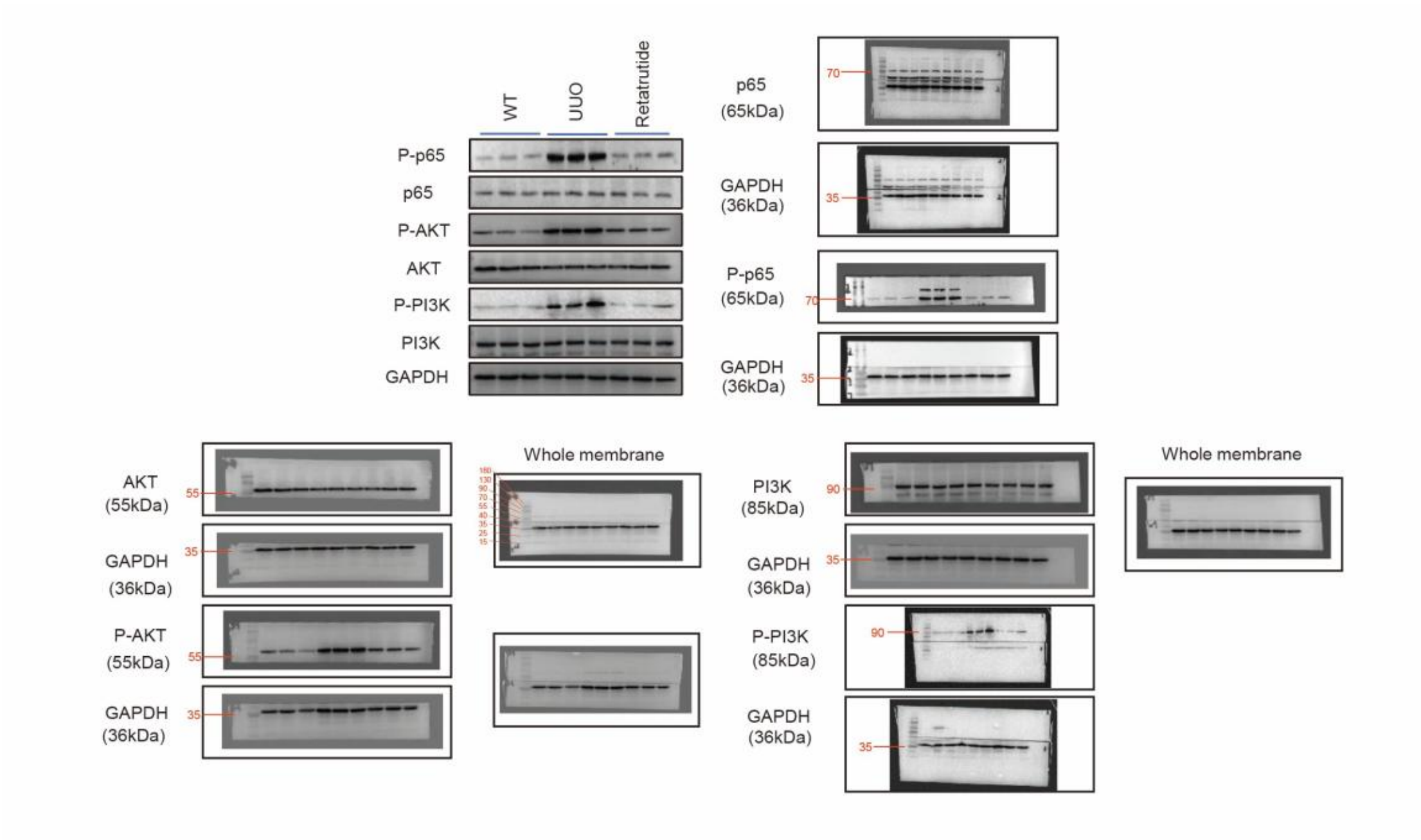

Data S4: Western blot original data for Figure 3G.

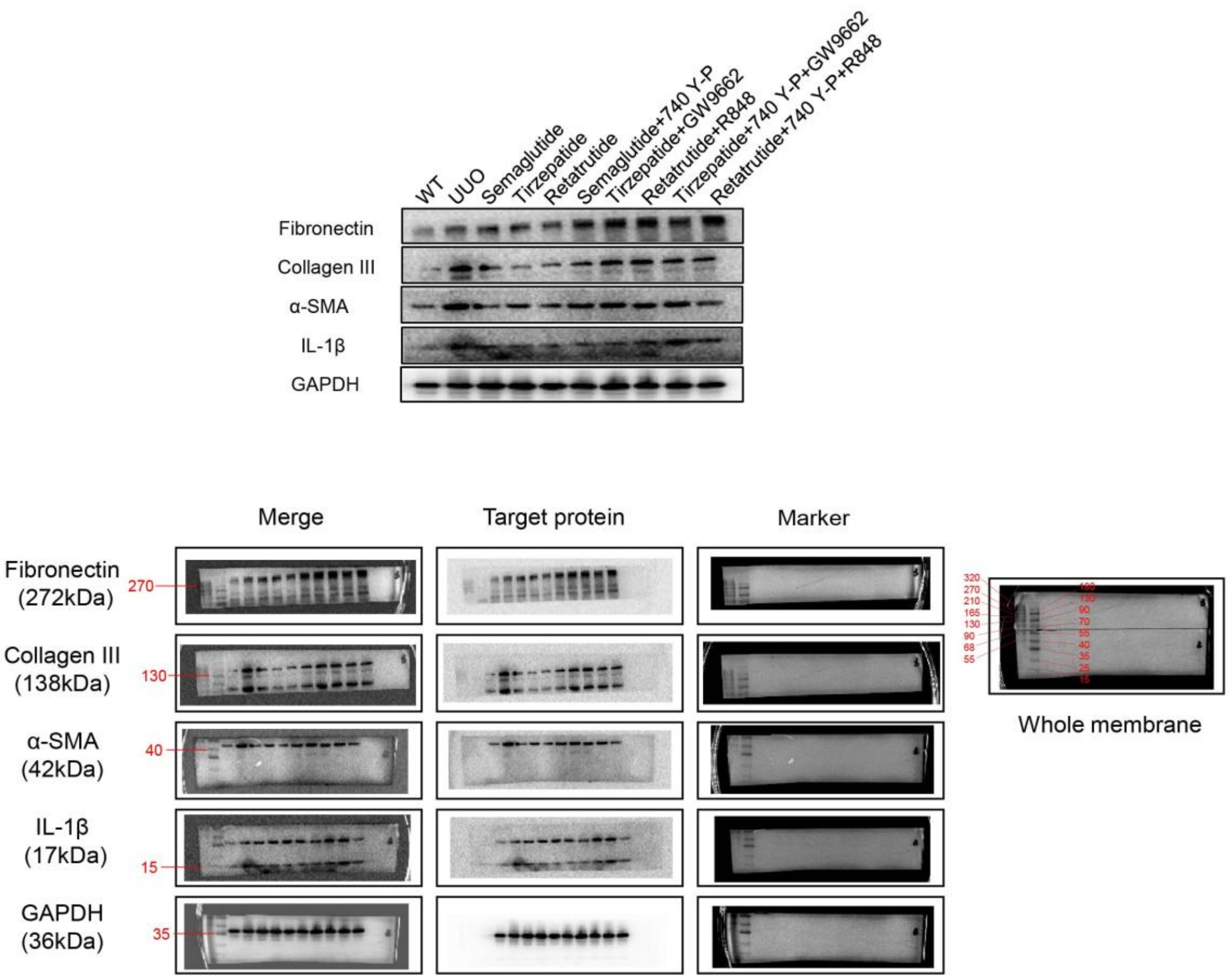

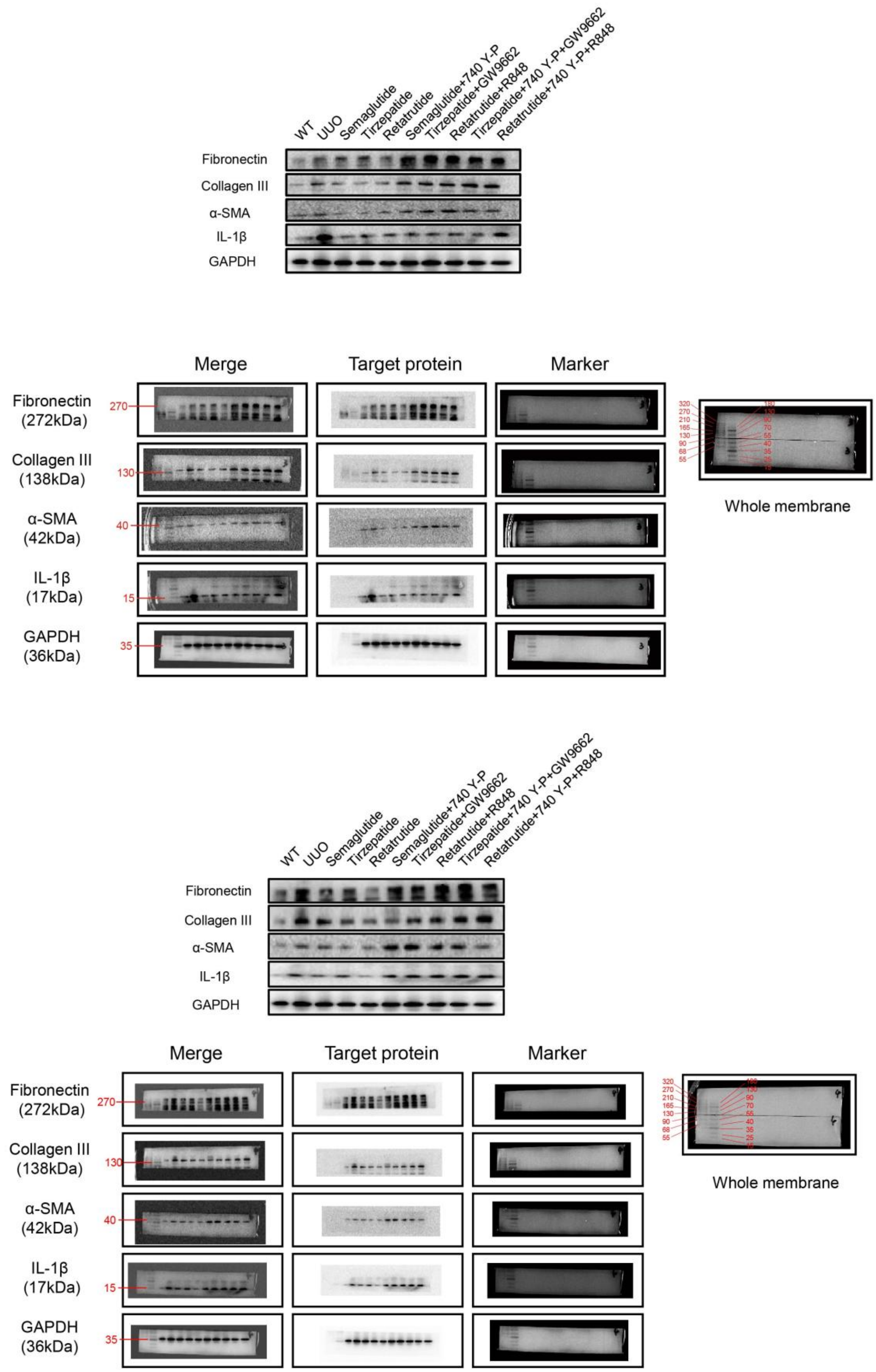

Data S5: Western blot original data for Figure 4H. (Three independent replicates were performed)

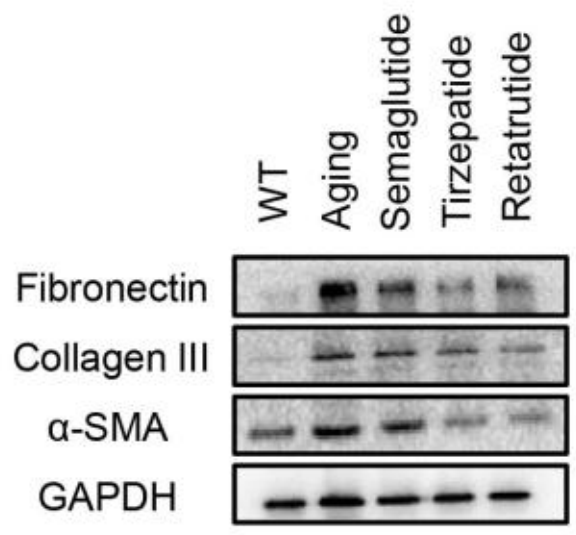

WT
Aging
Semaglutide
Tirzepatide
Retatrutide
Fibronectin
Collagen III
α-SMA
GAPDH

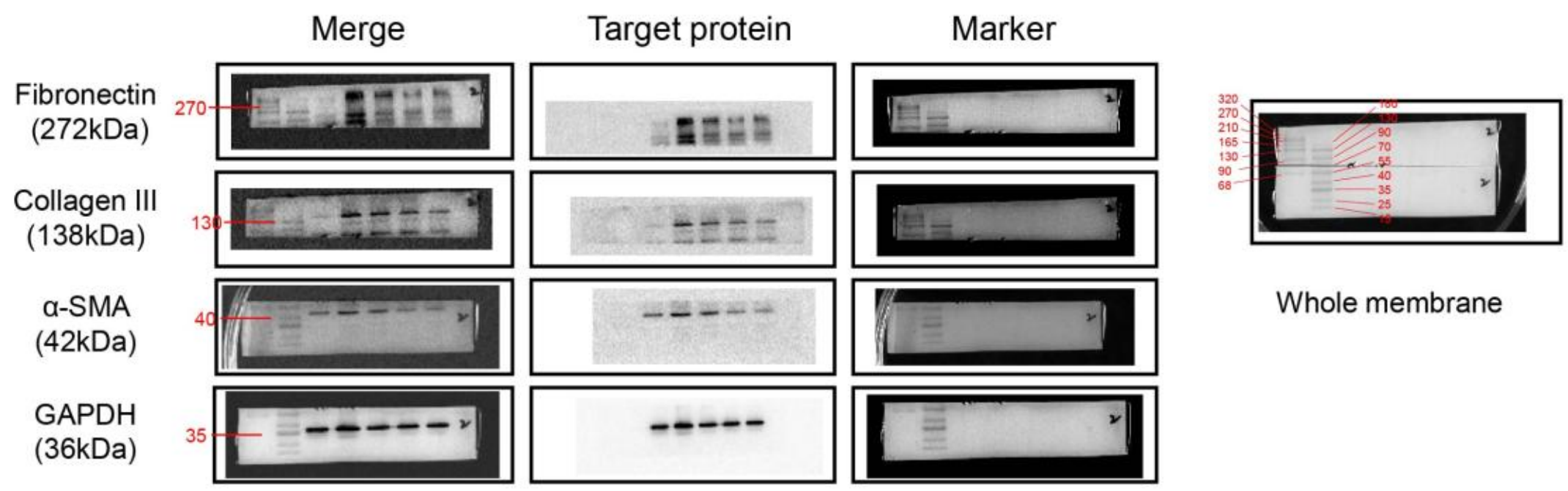

Merge
Target protein
Marker
Fibronectin (272kDa)
270
Collagen III (138kDa)
130
α-SMA (42kDa)
40
GAPDH (36kDa)
35
Whole membrane

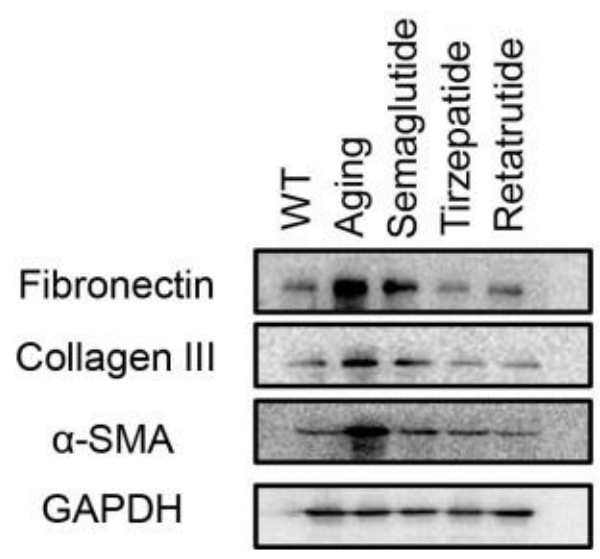

WT
Aging
Semaglutide
Tirzepatide
Retatrutide
Fibronectin
Collagen III
α-SMA
GAPDH

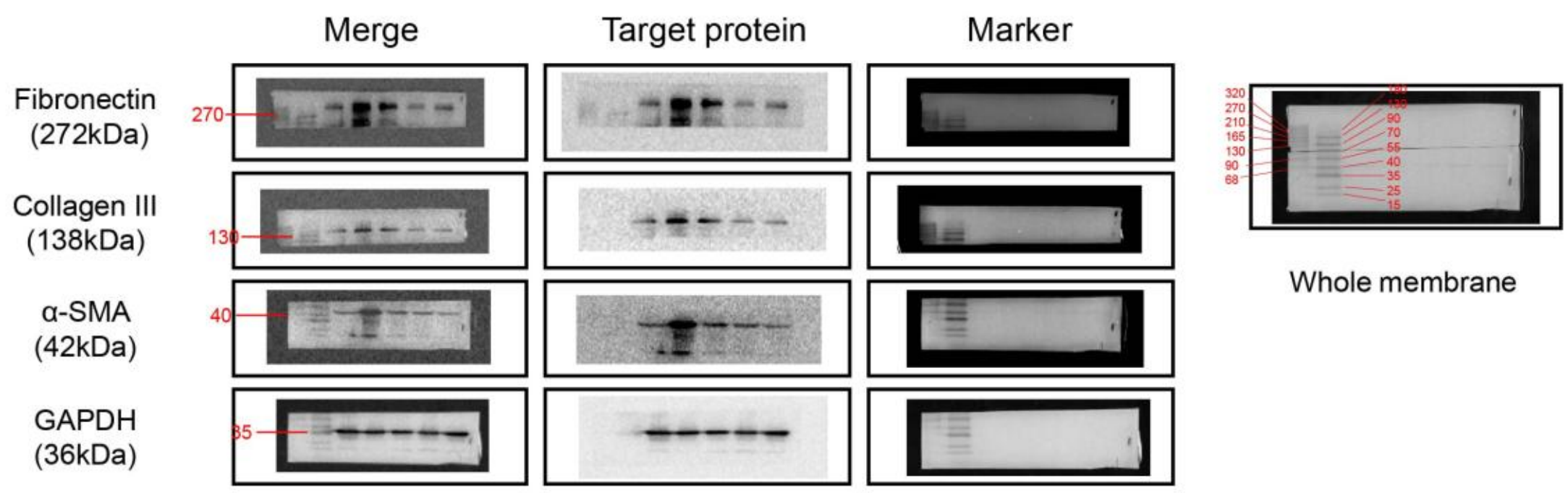

Merge
Target protein
Marker
Fibronectin (272kDa)
270
Collagen III (138kDa)
130
α-SMA (42kDa)
40
GAPDH (36kDa)
35
Whole membrane

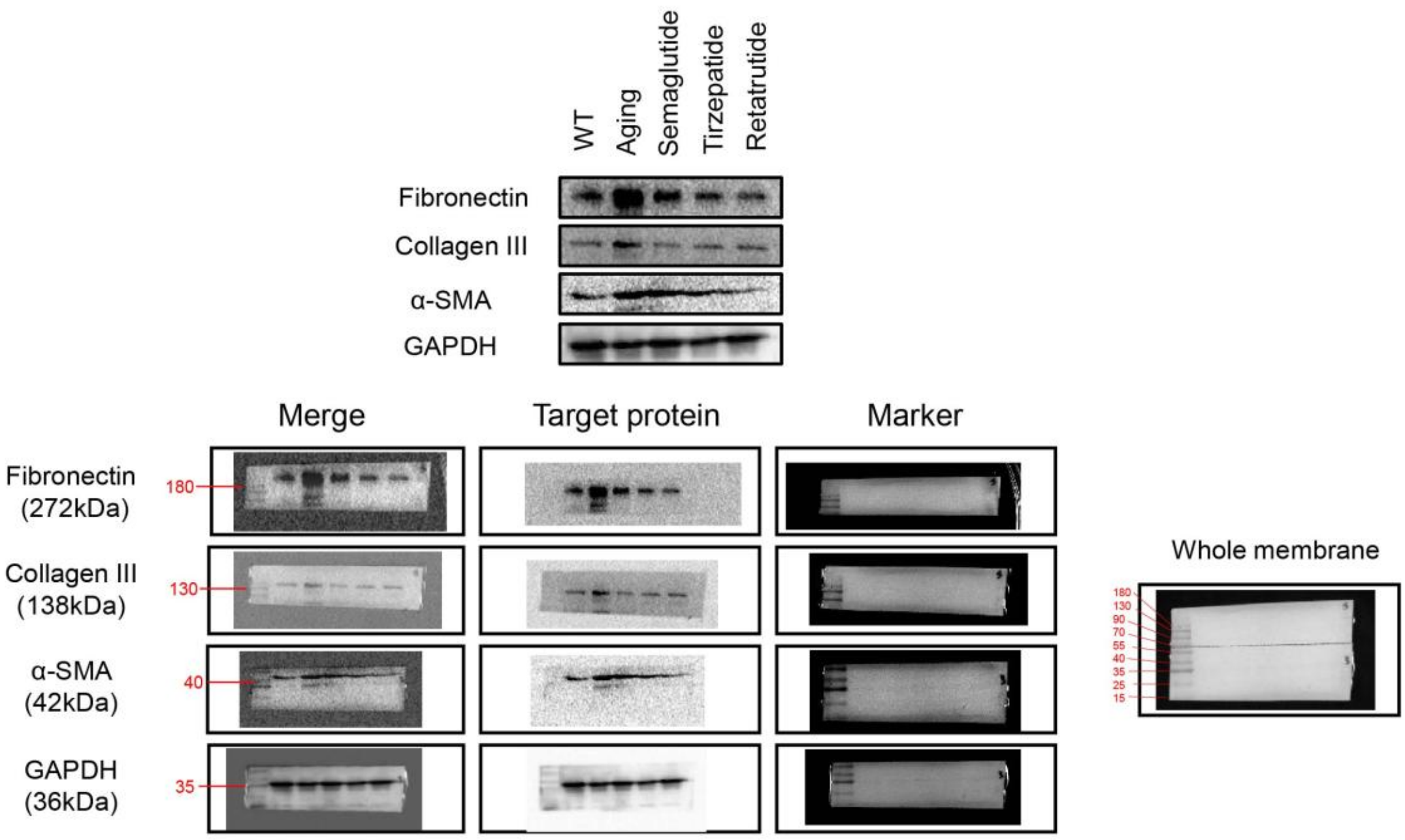

Data S6: Western blot original data for Figure 6G. (Three independent replicates were performed)

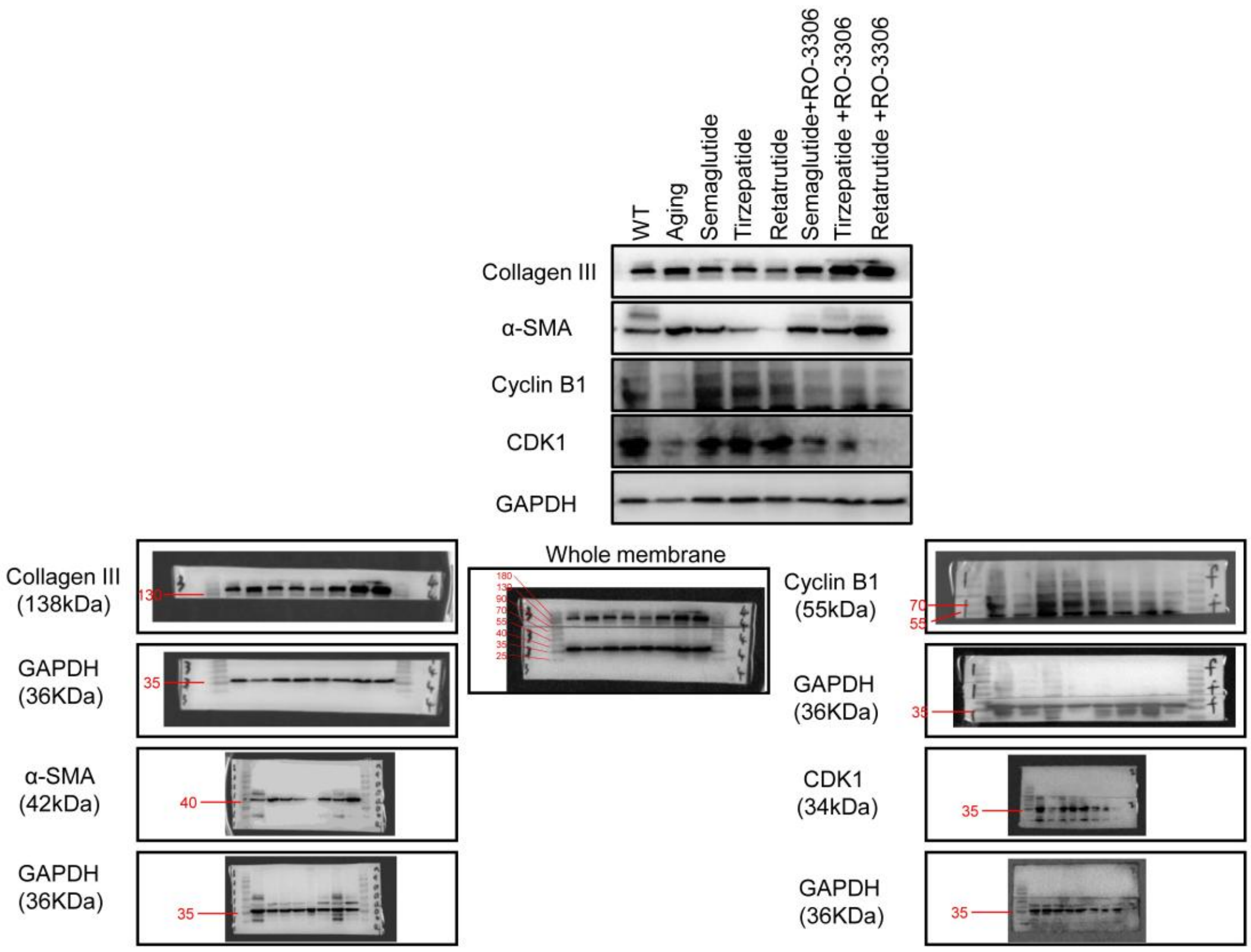

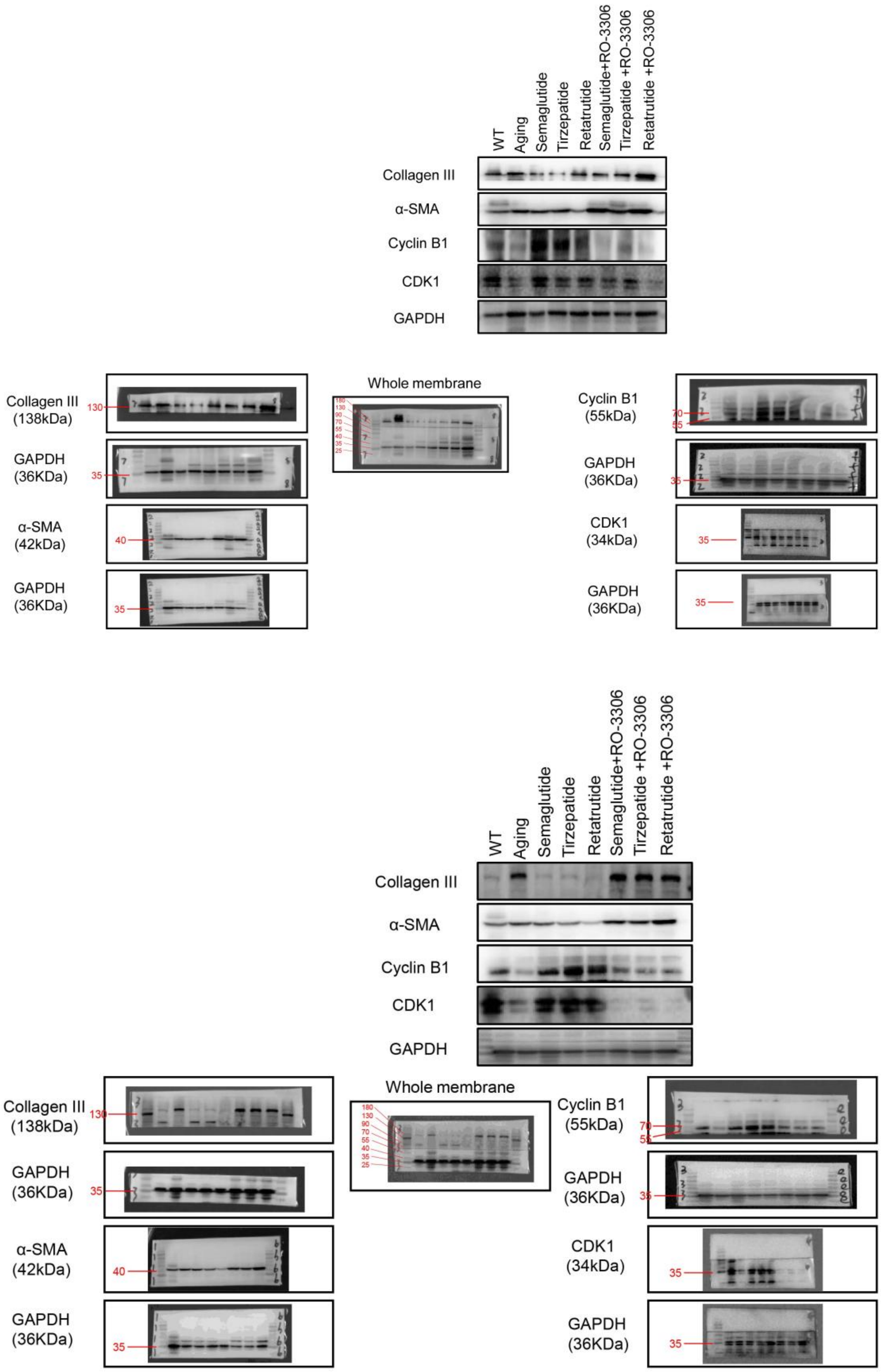

Data S7: Western blot original data for Figure 7H. (Three independent replicates were performed)

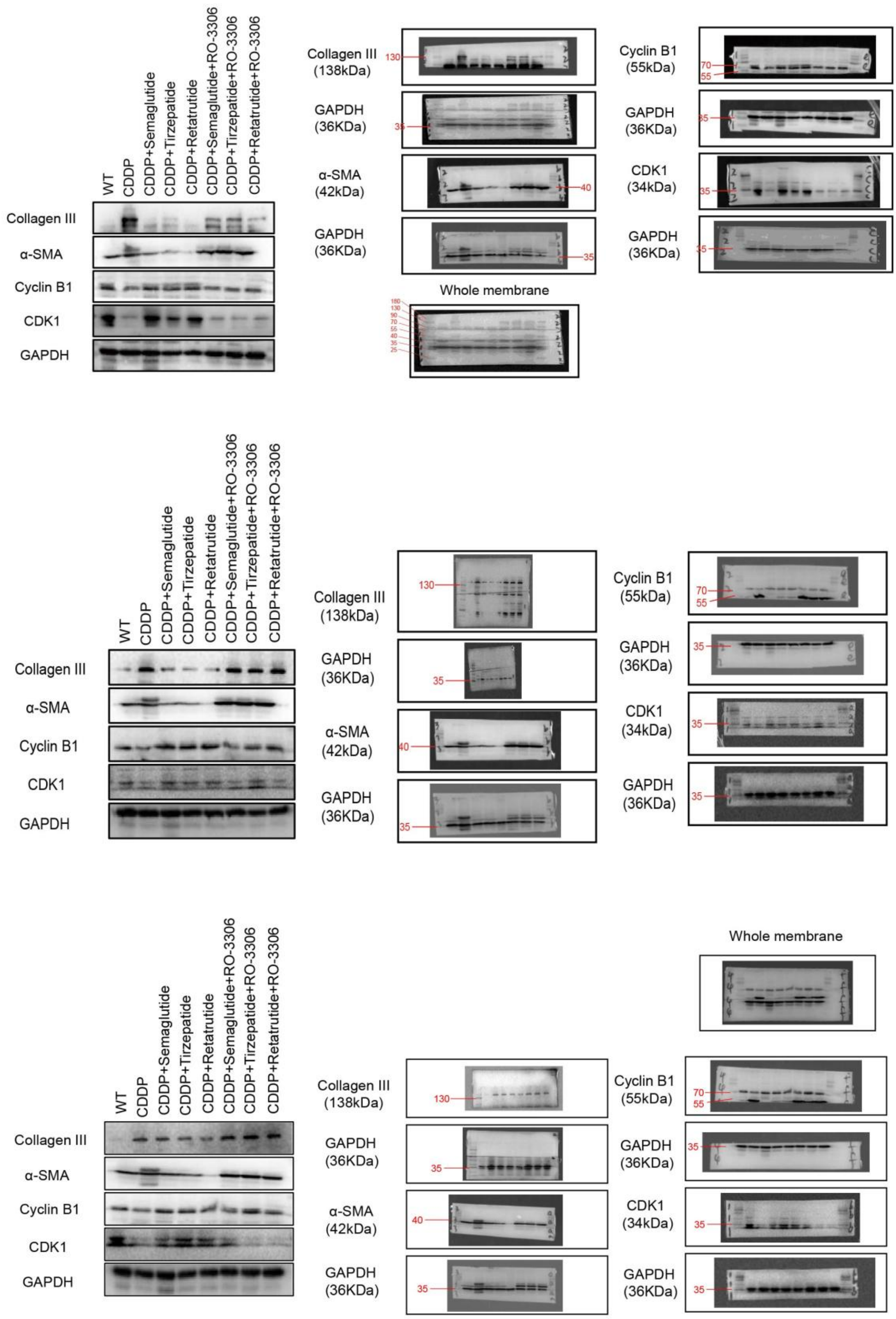

Data S8: Western blot original data for Figure 8C. (Three independent replicates were performed)
